# Telomeric RNAs are essential to maintain telomeres

**DOI:** 10.1038/ncomms12534

**Published:** 2016-08-17

**Authors:** Juan José Montero, Isabel López de Silanes, Osvaldo Graña, Maria A. Blasco

**Affiliations:** 1 Telomeres and Telomerase Group, Molecular Oncology Programme, Spanish National Cancer Research Centre (CNIO), 28029 Madrid, Spain; 2 Bioinformatics Group, Structural Biology and Biocomputing Programme, Spanish National Cancer Research Centre (CNIO), 28029 Madrid, Spain

## Abstract

Telomeres are transcribed generating long non-coding RNAs known as TERRA. Deciphering the role of TERRA has been one of the unsolved issues of telomere biology in the past decade. This has been, in part, due to lack of knowledge on the TERRA loci, thus preventing functional genetic studies. Here, we describe that long non-coding RNAs with TERRA features are transcribed from the human 20q and Xp subtelomeres. Deletion of the 20q locus by using the CRISPR-Cas9 technology causes a dramatic decrease in TERRA levels, while deletion of the Xp locus does not result in decreased TERRA levels. Strikingly, 20q-TERRA ablation leads to dramatic loss of telomere sequences and the induction of a massive DNA damage response. These findings identify chromosome 20q as a main TERRA locus in human cells and represent the first demonstration in any organism of the essential role of TERRA in the maintenance of telomeres.

## Abstract (web summary)

The telomeric long-non coding RNA, TERRA, has been proposed in the past to modulate different telomeric functions based on *in vitro* studies. Here the authors show, using a genetic deletion approach, that TERRA is transcribed from the 20q subtelomere and that it is essential for telomere maintenance.

## Graphical abstract

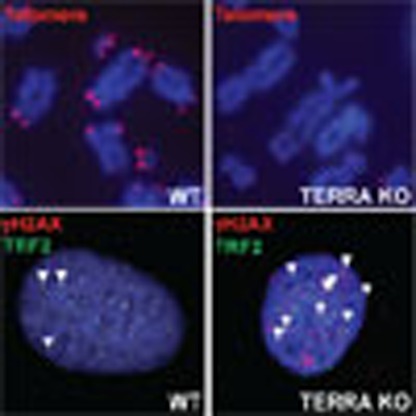

TERRA transcripts are nuclear long non-coding RNAs that are transcribed from the subtelomere towards the telomere^1^,^2^. They are transcribed by RNA polymerase II, giving rise to transcripts that contain UUAGGG-repeats, being the presence of this repeat their main features. They are also heterogeneous in size (0.2–10 kb in humans and mice) as indicated by the smear detected in TERRA northern blots^1^,^2^. On the other hand, detection of TERRA by RNA-fluorescence *in situ* hybridization (FISH) renders a clear spotted pattern in the nucleus and the number of spots varies in different cell types. Approximately 30% of these spots co-localize with telomeres^1^,^3^.

Lack of TERRA's subtelomeric sequence information has been an important shortcoming to understand the role of TERRA, as most the functional studies published to date are based on the use of the UUAGGG-tract to detect TERRA. Thus, TERRAs have been implicated in telomere protection^3^,^4^,^5^, heterochromatin formation^4^, telomere replication by sequestering hnRNPA1 from telomeres to allow RPA-to Pot1 switching, a process that it is altered in alternative lengthening of telomeres (ALT) cells lacking *ATRX*^6^,^7^, and in telomere elongation by homologous recombination through the formation of DNA-TERRA hybrids^8^,^9^. TERRAs are also proposed to bind the telomerase core components, TERC and TERT, but their role in telomerase function is unclear. On one hand, TERRA can inhibit telomerase activity *in vitro*^2^,^10^. On the other hand, in yeast, TERRAs are induced at short telomeres and form TERRA-TERC RNA clusters that co-localize with the telomere of origin during S phase, suggesting that TERRA may play a role in the spatial organization of telomerase activity at telomeres^11^.

In yeast, several TERRA transcription start sites have been identified at different chromosome ends. In the mouse, by using a genome wide RNA-seq approach, we recently identified that transcripts showing bona fide TERRA features mainly arise from the chromosome 18 subtelomere, and to a lesser extent from the subtelomere of chromosome 9 (ref. 3). Furthermore, we found that chromosome 18-TERRA associate in *trans* with the remaining telomeres and are important for telomere protection^3^. TERRA loci in human cells have been more elusive. First, a putative TERRA promoter was proposed to consist of a 61-29-37 repeat (a conserved region that contains three different repetitive DNA tracts of 61, 29 and 37-bp), which is present at 20 different chromosomes^12^. However, transcript sizes and their regulation were not identical to those of TERRA, suggesting that they may constitute a fraction of TERRA molecules^12^. More recently, a similar RNA-seq approach to that used by us in mice, identified 10 distinct human chromosome ends where putative TERRA transcription started as far as 5–10 kb away from the telomere, as well as eight additional chromosome ends where transcription started in close proximity to the telomere^13^. The authors, however, did not address whether these transcripts showed TERRA features, defined as the presence of UUAGGG-repeats within their sequence, their heterogeneity in size and the nuclear spotted pattern in which some spots co-localize with the telomere.

Here, we study whether these human transcripts have the above-mentioned TERRA features with the final goal of functionally deleting them to unveil the role of TERRA. We found that, out of the 18 proposed TERRA loci in humans, only transcripts arising from 20q and Xp loci have TERRA features. We then used the CRISPR-Cas9 technology to genetically delete these two potential TERRA loci in humans to study whether they are indeed the origin of TERRA. Only deletion of the 20q locus caused a dramatic decrease in TERRA levels, while deletion of the Xp locus did not result in decreased TERRA levels. Importantly, deletion of the 20q-TERRA locus leads to telomere shortening and telomere uncapping as indicated by increased telomere damage foci or TIFs. These unprecedented findings demonstrate that TERRA transcripts are essential for the maintenance of a functional telomere cap.

## Results

### Identification of the human TERRA locus

A number of subtelomeric transcripts identified in samples that underwent UUAGGG-transcript enrichment have been recently proposed to represent the human TERRA transcriptome, although further confirmation that these transcripts had TERRA features (see the first section) was lacking^13^. These RNAs arose from 10 distinct chromosome ends where transcription started as far as 5–10 kb upstream of the subtelomere-telomere boundary (referred here as ‘10-kb TERRA') and from 8 chromosome ends in which transcription started in close proximity to the telomere (referred here as ‘1-kb TERRA')^13^.

Here, we set out to study whether these subtelomeric transcripts have indeed TERRA features (see the first section), and if so, to generate functional knock-outs by using the CRISPR-Cas9 technology. With this approach, we aimed to unravel the role of TERRA in human cells.

**Figure 1:**
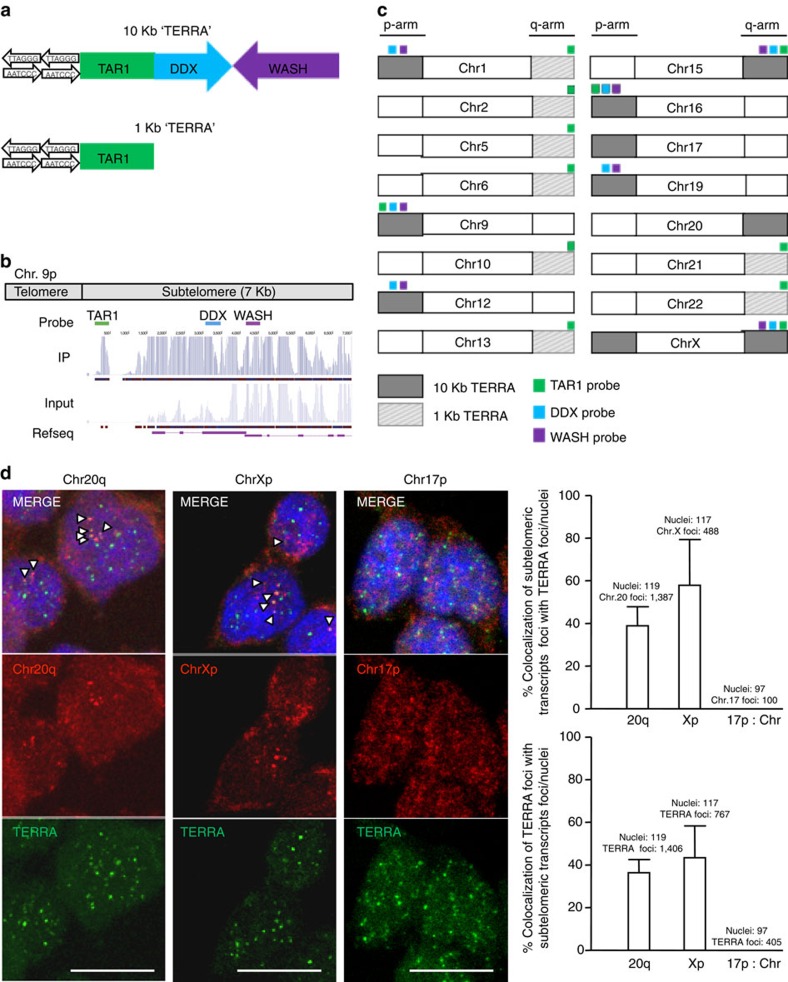
Transcripts arising from the subtelomere of chromosomes 20q and Xp co-localize with TERRA. (**a**) Schematic of the three motifs in the structurally conserved TERRA loci proposed in Porro *et al*.,^13^, the repetitive region TAR1 and the two lncRNA families, DDX11L and WASH. (**b**) Example of the position of the probes against the structurally conserved motifs in the subtelomere of chromosome 9p. The alignment of the RNA-seq reads obtained in a TERRA-IP compared with the input^13^ and an annotated Ref seq in this region is also shown. (**c**) Schematic of the position of TERRA loci proposed in Porro *et al*.,^13^ within all chromosomes. The 10 kb TERRA is coloured in grey and the 1-kb TERRA with stripes. The position of the probes against TAR1, DDX11L and WASH is also indicated in different colours. (**d**) Confocal microscopy images of double RNA-FISH using probes targeting either subtelomere 20q, Xp or 17p transcripts (red) and TERRA's telomeric tract (green). The representative images are the merge of four individual confocal images. Co-localization events were only counted as positive when detected in the individual confocal images. Arrowheads indicate real co-localization events detected in the individual confocal images. (Top graph) The percentage of co-localization of either subtelomere 20q foci or subtelomere Xp foci with TERRA foci with respect the total number of 20q or Xp foci is represented (mean±s.d., *n*=number of nuclei). Total number of nuclei and foci are also indicated. (Bottom graph) The percentage of co-localization of TERRA foci with either subtelomere 20q foci or subtelomere Xp foci with respect the total number of TERRA foci is also represented (mean±s.d., *n*=number of nuclei). Total number of nuclei and foci are also indicated. Scale bar, 10 μm.

First, we studied the genetic structure of the different identified sequences by aligning them against two different human assemblies, the UCSC human browser GRch37/hg19 and the subtelomeric specific assembly^14^. Bioinformatic analysis revealed that 83.3% of these transcripts arise from DNA regions that are structurally conserved. Thus, the ‘10-kb TERRA' loci contain three different conserved DNA elements, a repetitive region known as TAR1 (UCSC RepeatMasker track; Smit AFA, Hubley R, Green P. RepeatMasker Open-3.0, www.Repeatmasker.Org. 1996–2010) and two additional conserved DNA regions which encode for two long non-coding RNA families of pseudogenes derived from the primate genes WASH and DDX11 (refs 15, 16), respectively (Fig. 1a). From now on, we will refer to these three conserved DNA regions as TAR1, WASH and DDX regions/loci, and to the transcripts arising from these regions as TAR1, WASH and DDX transcripts. The ‘1-kb TERRA' contained only the TAR1 repetitive region (Fig. 1a). Surprisingly, the DDX region is transcribed in the opposite direction from what expected for a TERRA transcript^15^,^16^ (TERRA is transcribed from the subtelomere towards the telomere)^1^, thus suggesting that they are not likely to correspond to TERRA (Fig. 1b). Thus, transcripts arising from the subtelomeres of chromosomes 1p, 9p, 12p, 15q, 16p, 19p, Xq are unlikely to be bona fide TERRAs.

Nevertheless, we designed RNA-FISH and northern blot probes against the transcripts arising from each of the DNA structurally conserved regions, TAR1, DDX and WASH (Fig. 1c) and compared their signals to that of a probe detecting the TERRA's-UUAGGG-tract, as it is the TERRA main feature (see the first section). Sequence degeneration at subtelomeres allowed us to simultaneously detect transcripts arising from various subtelomeres (see probe specificity in Supplementary Tables 1–4). In double RNA-FISH experiments, we failed to see co-localization of sense (S) and antisense (AS) probes against TAR1, DDX and WASH RNAs with transcripts detected with the TERRA's-UUAGGG probe (Supplementary Fig. 1A). Sense and antisense probes were used since TAR1, DDX and WASH transcripts are not transcribed with the same orientation. Moreover, probes against TAR1 transcripts rendered a strong cytoplasmic signal, in contrast to the known nuclear localization of TERRA transcripts (Supplementary Fig. 1A). Of note, in the case of the DDX locus, whose transcripts are transcribed in the opposite direction than what is expected for a TERRA transcript, we failed to see co-localization with TERRA when using either the sense or the antisense DDX probes. As control, RNAse treatment erased all these different RNA-FISH signals confirming the specificity of the different probes to defect RNA and not DNA (Supplementary Fig. 1B). By using the northern blot analysis, we confirmed that transcripts arising from the TAR1, DDX and WASH loci did not show the expected TERRA pattern (Supplementary Fig. 1C). Furthermore, we did not detect any specific signal from the TAR1 probe, only the unspecific cross-hybridization with the 18S and 28S rRNAs (Supplementary Fig. 1C).

Next, we took advantage of the known effect of DNA methylation on regulating human TERRA levels to evaluate TERRA features in WASH, TAR1 and DDX transcripts. In particular, DNA demethylating agents are known to increase human TERRA levels^12^. To this end, we studied TERRA levels in the HCT116, Hela and U2OS human cell lines before and after treatment with the demethylating agent 5′ azacytidine (Aza), as well as in HCT116 cells double deficient for DNMT1 and DNMT3b (double knockout, DKO cells) as a positive control^17^. In marked contrast to TERRA transcripts, which were upregulated on Aza treatment, we failed to see induction of DDX, WASH and TAR1 transcripts on Aza treatment (Supplementary Fig. 1C,D), indicating that they do not show the expected TERRA behaviour^12^.

As none of the transcripts arising from the DNA structurally conserved loci TAR1, DDX and WASH showed features of TERRA (see the first section), we next studied the TERRA properties in the transcripts arising from the remaining non-conserved loci located at chromosomes 17p, 20q and Xp, which also showed ‘read enrichment' in the described TERRA-IP^13^. Interestingly, in double RNA-FISH experiments, we found co-localization of transcripts arising from 20q and Xp subtelomeres with TERRA transcripts (detected with a probe against TERRA's-UUAGGG), suggesting that they could correspond to genuine TERRA transcripts (Fig. 1d). In particular, ∼40% of signal spots from the 20q locus co-localized with TERRA signals, reaching 60% in the case of the Xp locus (Fig. 1d, top graph). From the total TERRA spots, 40% co-localized with spots from either 20q or Xp loci (Fig. 1d, bottom graph). Transcripts arising from the 17p locus, however, did not show co-localization with TERRA transcripts (Fig. 1d), thus ruling out that they contribute to TERRA. Note that the co-localizations were counted as positive only when detected in the individual confocal layers (see graphs). Apparent co-localizations between Chr17-RNAs and TERRA in the representative images of Fig. 1d were not counted, as they are the result of a visual effect of image superposition of individual confocal layers. Real co-localization events for Chr20q and Xp-RNAs with TERRA are indicated with arrowheads and were detected in individual confocal layers (Fig. 1d). As control, RNAse treatment also erased these RNA-FISH signals, thus confirming the specificity of the different probes (Supplementary Fig. 1B).

### Transcripts arising from the 20q locus are bona fide TERRA

**Figure 2:**
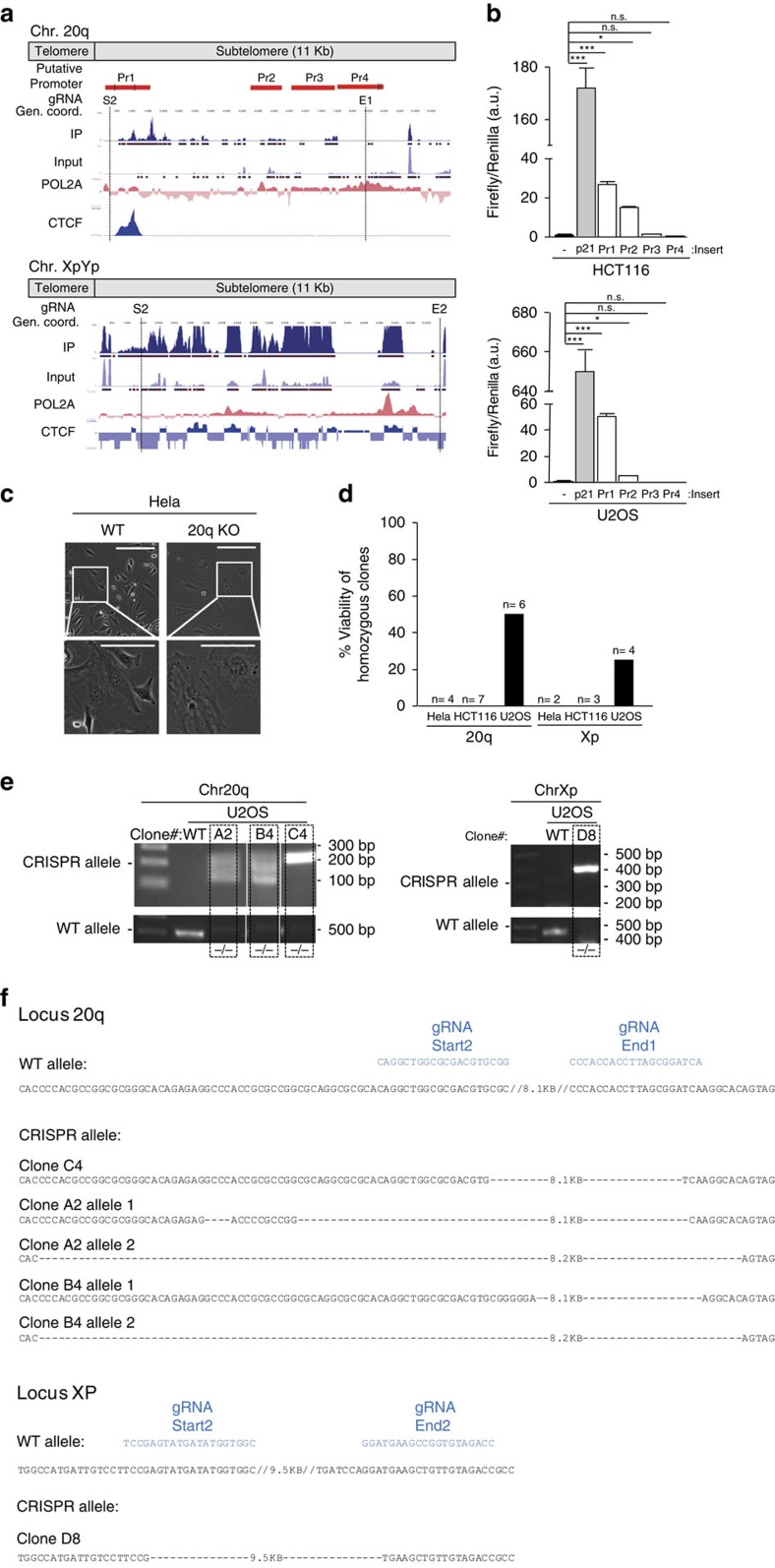
Deletion of the 20q-TERRA locus in different human cell lines using the CRISPR-Cas9 system. (**a**) Snapshot depicting, from top to bottom, putative promoter regions (Pr1, Pr2, Pr2 and Pr4), gRNA position, genomic coordinates, RNA-seq from TERRA IP and input^13^, and RNA polymerase 2A (POL2A) and CTCF ChIP-seq data. S2, E1, S2 and E2 are the names of the different gRNAs. (**b**) Graph shows the relative fold increase in firefly luciferase activity seen in the pGL3-containing promoter regions (Pr1-4) relative to the empty vector after normalization to renilla activity. (Mean values±s.e.m., *n*=3 independent experiments). p21 promoter serves as positive control. One-way Anova with the Dunnett's post test was used for statistical analysis (*P<0.05 and ***P<0.001). (**c**) Representative image of the HeLa clones homozygous for the 20q-TERRA deletion during expansion. Zoom areas are shown. Scale bar, 500 μm and (zoom) 250 μm. (**d**) Percentage of viability of the different homozygous clones for the 20q and Xp deletion on expansion of the three different cell lines. (**e**) Ethidium bromide gels showing the CRISPR allele for the deletion of the 20q and Xp loci detected by PCR in different clones of the U2OS cells. The white strips in the gels indicate the removal of irrelevant samples from that gel. (**f**) Schematic of the sequencing of the CRISPR allele for the deletion of the 20q (clones A2, B4 and C4) and Xp (clone D8) loci compared with the WT allele. Slashes (//) represent omitted DNA sequence. The size of the omitted sequence is shown. Dashes (-) represent the deleted sequence. The size of the deleted sequence is shown. gRNAs are shown in blue.

To further demonstrate that transcripts arising from 20q and Xp loci are genuine TERRA transcripts, we set to genetically delete the loci from which they are transcribed by using the CRISPR-Cas9 system in the HCT116, Hela and U2OS human cancer cell lines, as well as in the non-transformed IMR90 human fibroblast cell line. First, we performed a detailed transcriptional analysis of the regions to be deleted (Fig. 2a). Previously reported chromatin immunoprecipitation (ChIP)-seq data showed the presence of active RNA polymerase II and CTCF binding sites at both the 20q and Xp loci^14^. Thus, we assessed the presence of promoter activity in these regions by cloning different fragments upstream of the RNA-seq read enriched regions into promoter-free luciferase reporter vectors (Fig. 2a,b). We found a strong promoter activity for the 20q locus in the vicinity of the telomere (region Pr1) and to a lower extent in a region 4.7-kb away from the telomere (region Pr2) both in HCT116 and U2OS human cell lines (Fig. 2b). The p21 promoter was included as positive control (Fig. 2b). In the case of the Xp locus, we were unable to clone fragments of interest to assess its promoter activity owing to the highly repetitive content of this region. Collectively, these findings confirm strong transcriptional activity within the 20q locus.

Next, in order to delete these putative TERRA loci, we designed guide RNA (gRNAs) for both loci. In particular, we set to delete a 8.1-kb region in the 20q locus, which includes the promoter regions, and a 9.5-kb region in the Xp locus. Of note, these regions were the ones showing enrichment of RNA-seq reads in the previously described TERRA-IP^13^ (Fig. 2a). Using the online CRISPR designer tool^18^, two different gRNAs showing the lower off-target mutation score were selected for each of the flaking regions (gRNA-start 1 and start 2 for the region closer to the telomere and gRNAs-end 1 and end 2 for the region more distal from the telomere) (Supplementary Table 5). We tested the best combination of gRNAs by transfecting two plasmids, each of them containing the Cas9 and the GPF reporter but a different gRNA each, corresponding to each of the flanking regions (Supplementary Table 6). Two days post transfection, the presence of the deletion was studied by PCR using specific primers (Supplementary Table 7). For all the four human cell lines, we successfully detected different deletions of the 20q and Xp loci except for the Xp locus in HCT116 cells (Supplementary Fig. 2A). Next, we set up the conditions to select single clones with the deletion of interest. To this end, we transfected the most efficient combination of gRNAs for each flanking region. Each gRNA was within a plasmid containing the Cas9 and a green fluorescent protein (GFP) reporter. Two days post transfection, the top 10% GFP brightest cells were sorted by flow cytometry and seeded into 96-wells plates (1, 3, 5 or 10 cells per well). Two weeks after wells were checked for clonal cell expansion, and only those coming from single clones were genotyped by PCR (Supplementary Table 7). Clonal cell expansion was successful in all cell lines except for the non-transformed IMR90 fibroblasts (see the number and percentage of clones obtained for each genotype in Table 1). We then set to expand clones homozygous for the 20q and Xp deletions from Hela, HCT116 and U2OS cell lines. During expansion of Hela clones with the 20q-deletion, three out of four homozygous clones developed intense vacuolization and died (Fig. 2c,d). The remaining homozygous clones bearing either the 20q or the Xp deletion from the three cell lines (Hela, HCT116 and U2OS), except for few U2OS clones (see below), either did not grow or were found to be heterozygous for the deletion on the re-genotyping performed after expansion (Fig. 2d; Supplementary Fig.3A). Thus, we were unable to isolate viable clones with deletions in either 20q or Xp in both Hela and HCT116 cell lines, indicative of strong lethality of the deletions (Fig. 2d). Only in the case of U2OS cells, we were able to expand three clones out of six bearing the 20q-deletion (only six clones were expanded from the original 37 KO clones obtained; Table 1) and one clone out of four with the Xp deletion, which were confirmed to be homozygous both by PCR and by Sanger sequencing (clones A2, B4 and C4 for the 20q locus and clone D8 for the Xp) (Fig. 2e,f). The smear observed in the detection of the CRISPR-Cas9 alleles in clones A2 and B4 can be resolved in two distinct bands (Supplementary Fig. 3B) and it is due to two different deletions in each of the alleles of these clones within the 20q locus (see sequences displayed in Fig. 2f). Interestingly, we observed that U2OS cells display higher basal levels of TERRA compared with Hela and HCT116 cells (Supplementary Fig. 1B,C), which may explain the differential effects of TERRA abrogation on cell viability of the three different human cell lines studied.

**Figure 3:**
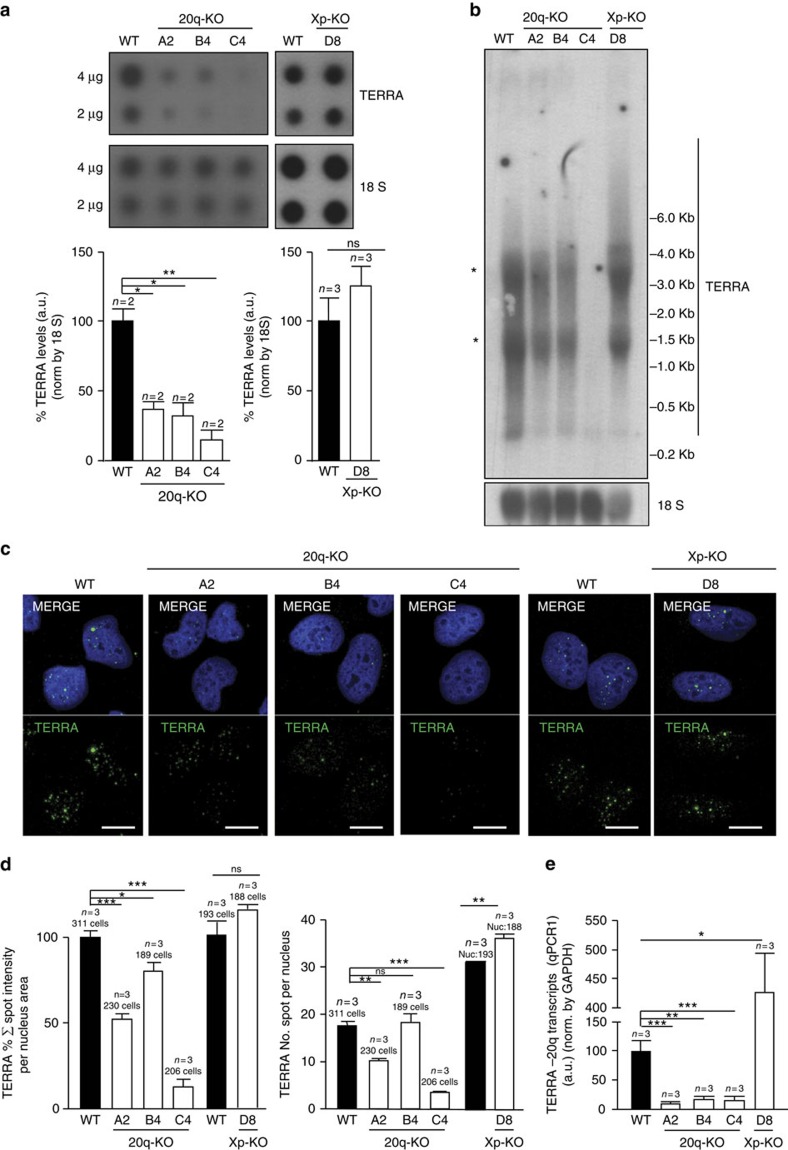
Deletion of the TERRA-20q locus dramatically affects TERRA expression. (**a**) RNA from the U2OS cells WT or from the three 20q-KO clones (A2, B4 and C4) and the Xp-KO (D8) clone was isolated and used for TERRA detection by RNA dot-blot with a probe against the TERRA-UUAGGG-tract; 18S serves as loading control. (Graph) TERRA quantification normalized by 18S (mean values±s.e.m.). (**b**) Northern blotting using 32P-dCTP-labelled probe against TERRA-UUAGGG-tract in the U2OS cells WT or KO for the 20q or Xp loci. 18S was included as a loading control. *Unspecific band due to cross-hybridization with rRNA 18S and 28S. (**c**) Representative confocal microscopy images of RNA-FISH against TERRA-UUAGGG-tract (green) in the U2OS cells WT and KO for the 20q (clones A2, B4 C4) and the Xp (clone D8) loci. Scale bar, 10 μm. (**d**) The graphs show (left) the quantification of the total spot intensity per nucleus normalized by nucleus area, (right) the total number of spots per nucleus in the three 20q-KO clones and in the Xp-KO clone D8 (mean values±s.e.m., *n*=3 independent experiments). (**e**) Detection of the 20q-TERRA transcripts by qPCR (primers were designed in the subtelomeric region). The percentage of enrichment of the 20q-TERRA transcripts in WT and in the 20q-KO clones (clones A2, B4 and C4) and in the Xp-KO (clone D8) normalized by GAPDH is shown. One-way Anova with Dunnett's post test was used for the statistical analysis of the 20q clones and the Student's *t*-test for the Xp clone (*P<0.05, **P<0.01 and ***P<0.001).

Next, we checked the expression of total TERRA levels by RNA dot-blot and northern blot in the individual clones deleted for the 20q and Xp loci. Deletion of the 20q locus resulted in a dramatic reduction in total TERRA levels in all three clones isolated from U2OS cells (see clones A2, B4 and C4 deleted for the 20q locus in Fig. 3a,b). In contrast, deletion of the Xp locus did not result in changes in total TERRA expression (Fig. 3a,b; Supplementary Fig. 4). The lack of TERRA downregulation as the result of the Xp deletion, may indicate that this is not a major locus for TERRA production in human cells. Alternatively, this may be related to the fact that the region deleted in the Xp locus was smaller than that deleted in the 20q locus, owing the impossibility to design gRNAs in the vicinity of the Xp telomere due to its highly repetitive nature.

Next, we confirmed TERRA downregulation owing to the deletion of the 20q locus but not of the Xp locus by several other independent techniques. In particular, RNA-FISH using probes that detect the TERRA's-UUAGGG-tract also demonstrated a dramatic decrease of the total TERRA spot intensity per nucleus in the 20q locus deleted clones but not in the Xp locus deleted one when compared with wild-type (WT) cells (in Fig. 3c,d, left graph). This was paralleled by a significant decrease in TERRA spots per nucleus in the 20q locus deleted clones but not in the Xp deleted one (Fig. 3d, right graph). Downregulation of TERRA levels in the 20q-deleted clones but not in the Xp deleted clone, was also confirmed by quantitative PCR (qPCR) (Fig. 3e; Supplementary Table 8).

To ascertain whether the changes in TERRA levels and in the phenotype observed (see below) could be due to the presence of CRISPR-Cas9 off-target mutations, we performed TIDE (Tracking of Indels by DEcomposition) analysis for each of the gRNAs used to generate the 20q-deletion (named Start1 and End1). We selected for study the five regions with the highest off-target mutation score but only the first four could be studied because of the impossibility to amplify the fifth region for each gRNA, probably owing to sequence differences between the U2OS genome and the human reference genome (GRCh38/hg38). The analysis was performed on DNA pools from either WT cells or cells bearing the 20q-deletion, named C3 (from which the clones A2, B4 and C4 were obtained). We did not find off-target mutations for any of the gRNAs except for the gRNA END1/off-target3 (Supplementary Fig. 5A). To verify whether this off-target mutation was present in the 20q-KO clones A2, B4 and C4, we carried out TIDE analysis on DNA from these clones. The 20q-KO clone A2 was free of gRNA END1/off-target3 but the clones B4 and C4 revealed 3–7% presence of off-target mutations (Supplementary Fig. 5B). It is unlikely that this was a real off-target mutation (vs the result of poor quality sequencing) because: (i) if an off-target mutation was present in these clones the percentage of off-targets should be 50 or 100% owing to the clonality of the cells, and (ii) comparison with DNAs from cells untreated with CRISPR revealed a similar percentage of off-target mutations (Supplementary Fig. 5B). Nevertheless, to confirm absence of off-target mutations, we performed T7-endonuclease digestion on DNA PCR amplified from the region of interest in both non-CRISPR-treated cells and from the CRISPR 20q-KO pool and clones (pool C3 and clones A2, B4 and C4). As a positive control, we used two identical DNAs except for a C to G mutation (DNAs were named C and G). As expected, two digestion bands were seen in the C+G digestion but no digested bands were observed neither in the C+C digestion nor in the cells that underwent the CRISPR deletion of the 20q locus (pool C3 and clones A2, B4 and C4) (Supplementary Fig. 5C). This assay confirms the absence of off-target 3 in the 20q-KO clones treated with the END1 gRNA. Note that the sequence alignment for the START2/off-target2 and for the END1/off-target4 regions was poor due to technical problems during sequencing because of the presence of long tracks of C or G within the sequences of these regions (Supplementary Figure 5A). Although we did not find off-targets mutations in the TIDE analysis of these regions, we further confirmed this finding with the T7-endonuclease assay (Supplementary Figure 5C).

Collectively, these data indicate that transcripts arising from the 20q locus are genuine human TERRA transcripts, which account for a large proportion of TERRA levels, while we failed to see TERRA downregulation when deleting the Xp locus. From now on we will refer to the 20q transcripts as ‘20q-TERRA' transcripts.

### 20q-TERRA locus deletion leads to loss of telomeric sequence

**Figure 4:**
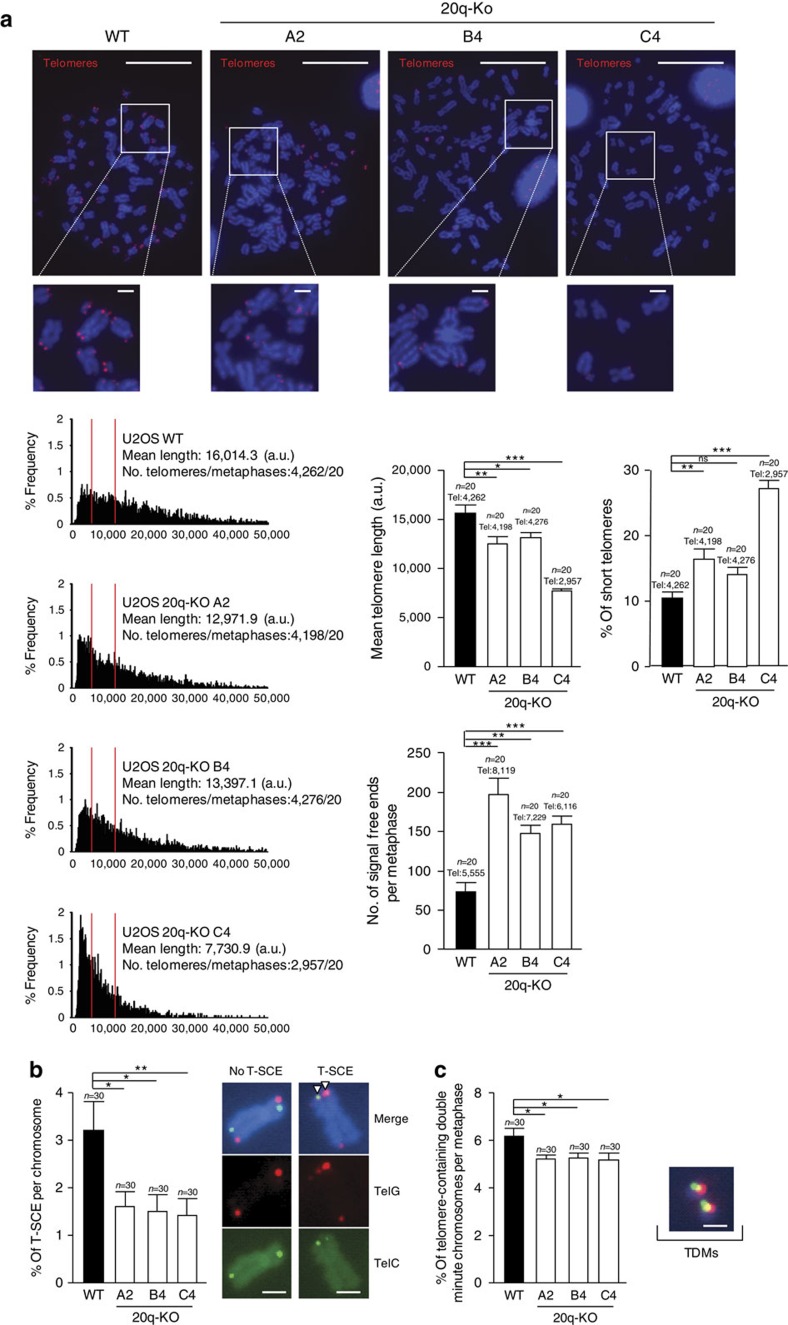
Deletion of the 20q-TERRA locus decreases telomere length and protection in U2OS cells. (**a**) Q-FISH images obtained from metaphases spreads from U2OS cells WT and KO for the Chr20q-TERRA locus (clones A4, B4 and C4). (Left graphs) Frequency graphs of telomere length (a.u.) distribution measured in WT and in the 20q-KO cells (clones A4, B4 and C4) from three independent experiments. The mean telomere length and the number of telomeres and metaphases analyzed is shown. The red lines are arbitrary lines placed in the exact same position in each frequency graph to visualize differences between the 20q-KO clones and the WT controls (right graphs) The mean telomere length, the percentage of short telomeres and the quantification of signal-free ends per metaphase are also represented. Short telomeres are considered those in the 10% percentile of the total telomere length distribution. Total number of metaphases used for the statistical analysis is indicated. Scale bar, 10 μm and (zoom) 1 μm. (**b**) WT and 20q-KO cells were analyzed for T-SCE events with G-rich (green) and C-rich (red) PNA probes. The fraction of chromosome ends with T-SCE obtained from three different experiments was quantified and graphed as the mean values±s.e.m., *n*=30 metaphases. The number of metaphases analyzed is shown. Only events in which interchange of both colours were quantified (see examples of no-T-SCE and T-SCE). The quantification was carried out by counting the number of events in the same chromosome or in different chromosomes and then normalizing it by the total number of chromosomes observed in each metaphase. Scale bar, 1 μm. (**c**) Quantification of DNA-containing double minute chromosomes (TDMs) in WT and 20q-KO cells from three different experiments (mean values±s.e.m., *n*=30 metaphases). An example of TDMs is shown. One-way Anova with Dunnett's post test was used for all statistical analysis (*P<0.05, **P<0.01 and ***P<0.001). Scale bar, 1 μm.

Identification of 20q as one of the major locus for human TERRA generation allows us to address the role of TERRA in telomere biology. Thus, we set to assess the impact of the 20q-TERRA locus deletion in telomere length and telomere protection. As shown in Fig. 4a, deletion of the 20q-TERRA locus resulted in a dramatic loss of telomeric sequences in all three clones (A2, B4 and C4) as seen by a significant decrease in individual telomere fluorescence intensity as determined by quantitative telomere FISH (Q-FISH) on single telomeres from metaphase spreads (see lack of detectable telomere signals in metaphasic chromosomes in Fig. 4a, and the dramatic change in the distribution of individual telomere signals), as well as by a significant increase in the percentage of signal-free ends per metaphase and in the percentage of short telomeres as determined by low telomere fluorescence (short telomeres are considered those in the 10% percentile of the total telomere length distribution) (Fig. 4a and graphs). Given that U2OS cells lack telomerase activity and elongate telomeres via a telomerase-independent mechanism that relies on homologous recombination, the so-called ALT^19^,^20^, we set to address whether the dramatic telomere shortening induced by deletion of 20q-TERRA locus was accompanied by decreased recombination events between telomeres. To this end, we measured the rates of telomeric sister chromatid exchanges (T-SCE) by using chromosome orientation FISH or CO-FISH^21^. Although we found T-SCE events in all the 20q-TERRA KO U2OS clones, their frequency was significantly decreased compared with the non-deleted U2OS controls (Fig. 4b). We also observed a significant decrease in telomere-containing double minute chromosomes on 20q-TERRA deletion in all three clones, a chromosomal aberration also associated with ALT (Fig. 4c)^22^. These results are in line with the proposed role of TERRA in telomere recombination^8^,^9^. However, the extremely short telomeres present in 20q-TERRA deleted cells, even in the presence of low but detectable telomere recombination events, suggests a more general role for TERRA in the maintenance of proper telomere homoeostasis.

**Figure 5:**
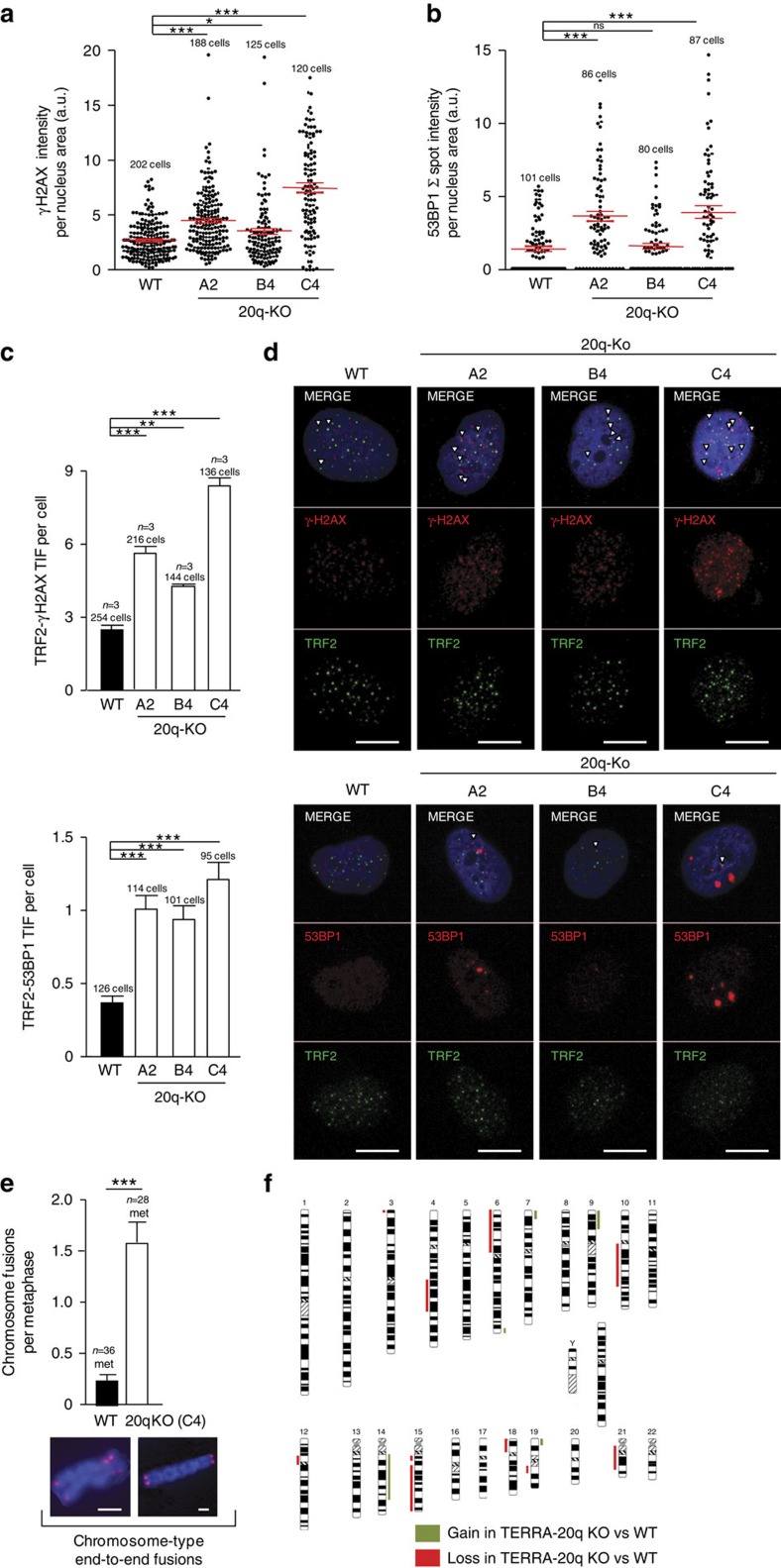
Deletion of the 20q-TERRA locus decreases telomere protection in U2OS cells. (**a**) Quantification of the total γH2AX signal per nucleus (mean values±s.e.m., *n*=number of cells) is shown. The total number of cells analyzed is indicated. (**b**) Quantification of the total 53BP1 spot signal per nucleus (mean values±s.e.m., *n*=number of cells is shown). The total number of cells analyzed is indicated. (**c**) Graphs showing the quantification of the co-localization (TIF) between TRF2 and either γH2AX or 53BP1 in WT cells and in all 20q-KO clones (mean values±s.e.m., *n*=3 independent experiments for γH2AX and *n*=number of cells for 53BP1) per cell is shown. The total number of nuclei analyzed is indicated. (**d**) Representative images of the average number of TIFs found on double inmunostain to detect the telomere protein TRF2 (green) and either the DNA damage markers phospho-Histone γH2AX or 53BP1 (red) in the U2OS cells WT or deleted for the 20q locus. Arrowheads indicate co-localization events. Scale bar, 10 μm. (**e**) Quantification of chromosomal end-to-end fusions in WT and in the 20q-KO cells from three independent experiments (mean values±s.e.m., *n*=metaphases). Examples of end-to-end fusions are shown as well. Scale bar, 1 μm. (**f**) Array-CGH analysis was performed on hybridization on the same membrane of DNA differentially labelled from WT and 20q-KO cells. The chromosomal gains and losses in 20q-KO cells normalized by WT cells are represented. The chromosomal gains are shown in green and in red the chromosomal losses. One-way Anova with Dunnett's post test was used for all statistical analysis except for the quantification of chromosomal fusions in which the Student's *t*-test was used (*P<0.05, **P<0.01 and ***P<0.001).

Next, we addressed the effects of TERRA deletion on telomere protection and chromosomal instability. First, we assessed telomere protection by determining the presence of either γH2AX or 53BP1 DNA damage foci at telomeres (the so-called telomere induced foci or TIFs). To this end, we performed double immunofluorescence with either γH2AX or 53BP1 antibodies to detected DNA damage foci combined with TRF2 immunofluorescence to detect telomeres. We found a significant increase in total DNA damage as indicated by increased γH2AX and 53BP1 fluorescence levels in all three clones except for the clone B4 in the case of 53BP1 (Fig. 5a, b). Interestingly, in all three clones and using both γH2AX and 53BP1, we found a significant increase in telomere damage foci or TIFs compared with WT controls (Fig. 5c,d). Finally, in agreement with short telomeres and increased telomere damage, phenotypes that were particularly severe in clone C4, we also found significantly increased end-to-end fusions in this clone (Fig. 5e). We further confirmed increased chromosomal instability in the 20q-TERRA KO C4 clone by comparative genome hybridization (CGH). We found dramatic genome reorganizations in the 20q-KO C4 cells compared with WT controls (Fig. 5f). In particular, we found chromosomal gains in chromosomes 7, 9, 14 and 19 along with large chromosomal losses in chromosomes 4, 6, 10, 15 and 21, including the complete loss of one of the arms in chromosomes 6 and 18, in the 20q-KO cells with respect WT cells. Some other smaller losses were also detected in chromosomes 3, 12, 18 and 19 (Fig. 5f). A complete list of the genomic coordinates of the different chromosomal gains and losses can be found in Supplementary Table 9.

Finally, to ascertain whether TERRA deletion led to changes in the telomere binding proteins or shelterins, we determined the amounts of TRF1 and TRF2 telomere binding proteins at telomeres by either ChIP or immunofluorescence (Supplementary Fig. 6A,B). We observed a reduced abundance of both TRF1 and TRF2 at telomeres by ChIP in all 20q-KO clones compared with WT controls, which was more dramatic in the C4 clone, in agreement with shorter telomeres and higher chromosomal instability in this clone (Supplementary Fig. 6A). The 20q-KO C4 clone also showed a significant reduction in TRF1 and TRF2 fluorescence (Supplementary Fig. 6B), in agreement with increased telomere damage and telomere aberrations^23^.

### Transcript identity between 20q-TERRA- and Xp-RNAs

Given the fact that we observed co-localization of the transcripts arising from the Xp locus with TERRA, we next tested the possibility of sequence similarities between the 20q-TERRA transcripts and those arising from the Xp locus. To that end, we use the RNA-seq data from Porro *et al.*,^13^ to generate modelled-transcripts (see ‘Methods' section) for the 20q and Xp loci (Supplementary Fig. 7). We next performed pairwise alignments between the modelled-transcripts from each locus to calculate the percentage of identity between them (Supplementary Fig. 7; Supplementary Table 10). By doing this, we found 50–80% identity between some of the 20q and Xp modelled-transcripts (Supplementary Table 10). However, we cannot rule out that this transcript similarity is due to either the small transcript size or to the presence of repeats within the sequence of the modelled-transcripts (there are multiple annotated repeats within the transcribing regions of the modelled-transcripts showing higher identity) or to both (Supplementary Table 11). To counteract the transcript size effect, we bioinformatically generated one single-transcript for each of the 20q and Xp regions that encompasses all the previously modelled-transcripts for each region (see above and Supplementary Table 12). Next, we performed pairwise comparison between the single-transcripts corresponding to each region. As expected, the percentage of identity between the 20q and Xp single-transcripts decreased to <40%, thus confirming the dependency of the sequence identity with the transcript size (Supplementary Fig. 7D).

We also found that transcripts arising from the Xp locus are more abundant than those arising from the 20q-TERRA locus (Supplementary Fig. 8A). Interestingly, the transcripts arising from the 20q locus were dramatically increased on deletion of the Xp locus as determined with three different pairs of primers designed within the 20q locus (Supplementary Fig. 8B). This finding is consistent with a compensation by the 20q-TERRA transcripts on deletion of the Xp locus explaining the absence of effects on total TERRA levels on deletion of the Xp locus (Fig. 3a,b). Deletion of the 20q locus also increased the Xp transcripts, mainly in the region in which the Xp deletion was generated (primer Xp/qPCR2) but not in the Xp region that was not deleted (primer Xp/qPCR1) (Supplementary Fig. 8B). Although these findings suggest a direct influence of the transcription of the 20q-TERRA locus on the Xp locus and vice versa, with the current Xp CRISPR deletion in the clone D8 we cannot conclude that the transcripts arising from the Xp locus are genuine TERRA transcripts. Additional studies will be needed to understand whether the transcripts arising from the Xp contribute to TERRA.

## Discussion

Here, we identified a major locus for human TERRA transcription at the subtelomere of the chromosome 20q. The genetic deletion of this locus allowed us to demonstrate for the first time in any organism the essential role of TERRA in the maintenance of functional telomeres.

In a previous report, eighteen different human subtelomeres were proposed to contain TERRA loci, although the authors did not study whether these transcripts had indeed TERRA features^13^. Here, we found that only two out of the eighteen loci have TERRA features, in particular, those corresponding to 20q and Xp subtelomeres. Furthermore, on genetic deletion of these loci by using CRISPR-Cas9, only the 20q locus was confirmed to be a bona fide TERRA locus since it resulted in almost complete abrogation of TERRA expression. Failure to decrease TERRA levels on Xp deletion may be explained by the impossibility to delete the entire Xp locus due to the highly repetitive nature of this region, or may indicate that this is not a bona fide TERRA locus. Alternatively, our finding that 20q-TERRAs are increased on deletion of the Xp locus, may also explain the fact that Xp deletion did not result in decreased TERRA levels. In any case, the fact that deletion of the 20q locus results in dramatically reduced TERRA levels demonstrates that this locus is a major TERRA locus in human cells.

Interestingly, the finding of one (or two at most) TERRA locus in human cells resembles the situation recently described by us in the case of mouse TERRA^3^. In particular, by using a similar approach to that used in humans (RNA-seq following UUAGGG-transcript enrichment), we identified mouse transcripts arising from thirteen different chromosome ends but only those transcribed from the subtelomere of chromosome 18 and 9 were confirmed to be bona fide TERRA transcripts, being the chromosome 18 locus responsible for the majority of TERRA transcripts^3^. Thus, both in human and mouse, TERRAs are transcribed from one or two subtelomeres, a finding that was not anticipated since it was widely accepted that TERRA were arising from all chromosome ends. It remains to be deciphered whether TERRA transcripts play different roles depending on the locus of origin.

Importantly, here we describe the first genetic deletion of TERRA performed in any organism. The findings are striking as they clearly demonstrate an essential role of TERRA in cellular viability as well as in telomere maintenance and telomere capping. In particular, the impossibility to expand most of the homozygous clones for the 20q-deletion in three independent human cell lines (Hela, HCT116 and U2OS) suggests an essential role of TERRA for cell survival in the majority of cell lines studied here. Only the U2OS cell line, which expresses higher basal levels of TERRA (this paper; refs 7, 8), could bear the 20q-deletion suggesting that the cell needs to maintain a minimum amount of TERRA levels to survive. Importantly, we found a dramatic loss of telomeric sequences in U2OS cells on deletion of the 20q-TERRA locus, indicating that TERRA is essential for telomere maintenance. This dramatic telomere shortening was accompanied by telomere uncapping as indicated by increased telomeric damage and increased telomere fusions. Interestingly, U2OS cells are known to maintain telomeres by homologous recombination, a process in which TERRA has been suggested to participate^7^,^8^,^9^. In line with this, we found detectable but significantly decreased telomere recombination frequencies as indicated by T-SCE frequency. Together, these results indicate an essential role for TERRA in cellular viability and in the maintenance of telomeres, even in cells that maintain telomeres by recombination.

Interestingly, deletions and amplifications of the 20q subtelomere have been found in patients with different types of hematopoietic malignancies and with mental retardation^24^,^25^,^26^,^27^. Although some of these deletions also include coding genes, it is tempting to speculate a specific role of the 20q-TERRA on these malignancies. Finally, the finding of the human 20q-TERRA locus described here warrants future studies to analyze of a possible association of 20q-TERRA to these and other human diseases.

## Methods

### Cells and treatments and transfection

Human HCT116, HeLa, U2OS (ATCC) and HCT116 DKO^17^ were cultured according to the ATCC's recommendations. 5′ Azacytidine treatment was performed at 1 mM for 3 days. Plasmids were transfected with Lipofectamine 2000 (Thermo Scientific) except for the IMR90 cell line that was electroporated using the Neon Transfection System (Invitrogen), in both cases following the manufacturers' protocol.

### RNA-deep sequencing alignment

Raw data from RNA-seq samples was downloaded from GEO (GSE56727) and analyzed with the nextpresso pipeline, as follows: sequencing quality was checked with FastQC (http://www.bioinformatics.babraham.ac.uk/projects/fastqc/). Due to a decrease in nucleotide quality at the end of the reads across the samples, the last 31 nucleotides were trimmed. Reads were then aligned to the human genome (GRCh37/hg19) with TopHat-2.0.10 (ref. 28), using Bowtie 1.0.0 (ref. 29) and Samtools 0.1.19 (ref. 30), allowing 4 mismatches and 5 multihits. In a similar way, reads were aligned to the human subtelomeric reference sequences downloaded from the H.C. Riethman lab web page (http://www.wistar.org/lab/harold-c-riethman-phd/page/subtelomere-assemblies).

### RNA-FISH

Cells grown on poly-L-lysine-coated coverslips (Becton Dickinson) were placed in cytobuffer (100 mM NaCl, 300 mM sucrose, 3 mM MgCl_2_, 10 mM pipes pH 6.8) for 30 s, washed in cytobuffer with 0.5% Triton X-100 for 30 s, washed in cytobuffer for 30 s and then fixed for 10 min in 4% paraformaldehyde in phosphate-buffered saline (PBS). The cells were dehydrated in 70, 80, 95 and 100% ethanol, air dried and hybridized overnight at 50 °C with RNA probes in hybridization buffer (2 × sodium saline citrate (SSC)/50% formamide). Coverslips were washed two times for 15 min in hybridization buffer at 53 °C, two times for 10 min in 2 × SSC at 53 °C, 10 min in 1 × SSC at 53 °C, 5 min in 4 × SSC at room temperature, 5 min in 4 × SSC containing 0.1% Tween-20 and DAPI (Molecular Probes) at room temperature and 5 min in 4 × SSC at room temperature. Signals were visualized in a confocal ultraspectral microscope SP5-WLL (Leica). TERRA signal was quantified using the Definiens Developer XD.2 software RNA-FISH probes were generated from PCR products by *in vitro* transcription (Ambion) using Cy3-labelled CTP (Amersham); primers available in Supplementary Table 13. Quantification of the percentage of co-localization in double RNA-FISH was done by counting the average number of co-localization events detected in the individual confocal layers with respect to the total number of foci per nucleus arising from the RNA to be quantified. The percentage of co-localization was then represented (Fig. 1d).

### Northern blot and dot-blot

Northern blot and dot-blot analyses were performed using standard protocols. TERRA probe was obtained from a 1.6-kb (TTAGGG)*n* cDNA insert excised from pNYH3 (kind gift from T. de Lange, Rockefeller University, NY, USA). Northern was normalized using 18S probes and quantified using ImageJ. Probes against subtelomere transcripts were prepared from PCR products from total cDNA (see primers used in Supplementary Table 13). The probes were labelled using the commercial Random Prime Labelling System Rediprime II (Amersham).

### Plasmids and reporter assays

For construction of the reporter plasmid, PCR products were prepared with primers spanning the promoter regions of interest (Supplementary Table 14) and cloned into the plasmid pGL3-basic (Promega). Firefly and Renilla Luciferase activities were measured in cells harvested 2 days after transfection with the Dual Luciferase Reporter Assay System (Promega), following the manufacturer's instructions.

### Generation of TERRA KO clones using the CRISPR-Cas9 system

The sequence for each gRNA (Supplementary Table 5) were obtained using a online CRISPR Design Tool^18^ and those with the best score for ruling out off-target mutations were chosen. Each gRNA was cloned into the plasmid pSpCas9(BB)-2A-GFP (PX458) (Gift from Feng Zhang) (Addgene plasmid #48138) using the protocol described in Hsu *et al*.,^18^. Cells were transiently co-transfected with two of this plasmid containing each one the Cas9 and a specific gRNA. Single cell clones were obtaining by sorting. To this end, two days after transfection cell were trypsinized and washed with dPBS, 1, 3, 5 or 10 cells of the 10% GFP brightest ones were sorted using the FACS ARIA IIU (Becton Dickinson) into 96-wells plates. Two weeks later, wells were checked for monoclonal cell expansion and that cells were genotyped by PCR. Homozygous monoclonal cell lines were expanded. PCR primer can be found in Supplementary Table 7.

### PCR and Sanger sequencing

For sequencing, samples were amplified using the GoTaq polymerase (Promega) and cloned into the commercial plasmid pCR2.1-TOPO (Invitrogen) following the manufacturer's instructions. Samples were sequences using the Sanger-style BigDye terminator chemistry on an ABI 3730 × l sequencer (Applied Biosystems).

### Telomere QFISH and chromosomal aberrations on metaphases

Colcemide (Gibco) was added to cells at a concentration of 0.1 μg ml^−1^ during 4 h. After hypotonic swelling in 0.03 M sodium citrate for 25 min at 37 °C, cells were fixed in methanol:acetic acid (3:1). After 2–3 fixative changes, the cell suspension was dropped onto clean, wet microscope slides and dried overnight. Then, slides were washed with PBS and fixed for 2 min in 4% formaldehyde. Slides were washed three more times with PBS and treated with pepsin (1 mg ml^−1^, pH 2) at 37 °C for 10 min. Formaldehyde fixation and washing steps were repeated, then slides were incubated in 0.4 M HCl for 10 min, rinsed in PBS, dehydrated in an ethanol series (70, 90 and 100%) and air dried. The Cy-3-labelled (CCCTAA)3 PNA probe (Panagene) was dissolved in hybridization buffer containing 70% formamide/10 mM Tris pH 7 and 0.25% (w/v) blocking reagent (0.5 μg ml^−1^) (Roche). The hybridization mixture was placed onto the slides and a coverslip was applied, followed by DNA denaturation (3 min, 80 °C). After hybridization (2 h, room temperature), slides were washed twice with 70% formamide/10 mM Tris pH 7.2 for 15 min, followed by a 5 min washing in 0.05 M Tris/0.15 M NaCl pH 7.5/0.05% Tween-20. Slides were dehydrated in an ethanol series and air dried. Finally, slides were counterstained with Vectashield containing DAPI (Vector Laboratories, Burlingame, CA). The cut-off used in the determination of the percentage of short telomeres was determined by averaging the results obtained after subtracting the 10% percentile from the mean telomere length value in the WT condition. For analysis of chromosomal aberrations, metaphases were analyzed by superimposing the telomere image on the DAPI image using TFL-telo.

### Immunofluorescence

Cells grown on poly-L-lysine-coated coverslips (Becton Dickinson) were placed in cytobuffer (100 mM NaCl, 300 mM sucrose, 3 mM MgCl_2_, 10 mM pipes pH 6.8) for 30 s, washed in cytobuffer with 0.5% Triton X-100 for 30 s, washed in cytobuffer for 30 s for and then fixed for 10 min in 4% paraformaldehyde in PBS. Cells were blocked with 100% FBS for 1 h at room temperature. Cells were incubated with primary antibody dissolved in Dako antibody Diluent (Dako) overnight in a humid chamber at 4 °C. Next day, coverslips were washed 3 times for 30 min in 1 × PBS containing 0.1% Tween-20. Cells were incubated with Alexa secondary antibody (Life Technologies, A11017) dissolved in Dako antibody Diluent (Dako) for 1 h in a humid chamber at room temperature. Cells were washed three times for 30 min in 1 × PBS. Samples were mounted in Prolong with Dapi (Invitrogen). Signals were visualized in a confocal ultraespectral microscope SP5-WLL (Leica). The following antibodies were used: anti-phospho-Histone γH2AX (05-636, Millipore) diluted 1:200, anti-53BP1 (NB-100-304, Novus) diluted 1:200, anti-TRF1 (TRF78, Abcam) diluted 1:200 and anti-TRF2 (clone 4A794, Millipore) diluted 1:200.

### CGH array

Oligo array-CGH analysis was performed using an Agilent SurePrint G3 Human CGH 60 K microarray (AMADID 021924 Agilent Technologies, Santa Clara, CA, USA) spanning the entire human genome at a median resolution of 41 kb. Totally, 500 ng of genomic DNA from the 20q-KO and WT cells were differentially labelled by random priming with Cy5-dCTP and Cy3-dCTP. The hybridization was carried out according to the manufacturer's protocol. Arrays were scanned using an Agilent DNA Microarray scanner G2565CA (Agilent Technologies). Microarray data were extracted and visualized using Feature Extraction software v10.7 and Agilent Genomic Workbench (AGW) software v7.0 (Agilent Technologies). Copy number altered regions were detected using the Aberration Detection Method 2 (ADM-2) algorithm set as 6, with a minimum number of three consecutive probes.

### Chromosome orientation FISH (CO-FISH)

Exponentially growing cells were sub-cultured in the presence of 5′-bromo-2′-deoxyuridine (BrdU; Sigma) at a final concentration of 1 × 10^−5^ M, and then allowed to replicate their DNA once at 37 °C for 24 h. Colcemide (Gibco) was added at a concentration of 0.1 μg ml^−1^ during the last 4 h. Cells were then recovered and metaphases prepared (see Q-FISH methods). The slides were treated with 0.5 mg ml^−1^ RNase A for 10 min at 37 °C, stained with 0.5 μg ml^−1^ Hoechst 33258 (Sigma) in 2 × SSC (0.3 M NaCl, 0.03 M sodium citrate) for 15 min at room temperature and then exposed to 365 nm UV light (Stratalinker 1800 UV irradiator) for 25–30 min. Enzymatic digestion of the BrdU/BrdC-substituted DNA strands with 3 U μl^−1^ of Exonuclease III (Promega) in buffer supplied by the manufacturer (50 mM Tris–HCl, 5 mM MgCl_2_ and 5 mM dithiothreitol, pH 8) was allowed to proceed for 10 min at room temperature. An additional denaturation in 70% formamide, 2 × SSC at 70 °C for 1 min was performed in order to ensure complete removal of the newly replicated bromo-substituted strands and followed by dehydration in a cold ethanol series (70, 85, 100%). A hybridization mixture containing 0.4 μg ml^−1^ of CY3-labelled PNA probe against the telomeric lagging strand (Panagene) in 30% formamide and 2 × SSC was applied to slides. Following 4 h of hybridization in a moist chamber at 37 °C, the slides were washed 5 times for 15 min each in 2 × SSC at 42 °C. The same hybridization step was performed with a hybridization mixture containing 0.4 μg ml^−1^ of Fluoresceine-labelled PNA probe against the telomeric leading strand (Panagene). Finally, slides were counterstained with Vectashield containing DAPI (Vector Laboratories, Burlingame, CA, USA). T-SCE events were counted when interchange of both probes was seen.

### Bioinformatic analysis of the probe specificity

The bioinformatic prediction of the regions amplified by the different primers to generate the probes was calculated using the *in silico* PCR tool from the UCSC human genome browser GRCh38/hg38. The homology of the best amplicon for each pair of primers was calculated using the NCBI nucleotide BLAST aligning the sequence of the first 15-kb from each subtelomere sequence obtained from the subtelomeric browser from Stong *et al*.,^14^ against all the possible amplicons sequence.

### Transcript identity studies

Reads from RNA-seq samples^13^ were aligned to the human subtelomeric reference sequences downloaded from the H.C. Riethman lab web page (http://www.wistar.org/lab/harold-c-riethman-phd/page/subtelomere-assemblies). To study transcript identity, alignments were used to obtain transcripts modelled by Cufflinks inside the chr.20q and chr.Xp subtelomeric regions of interest (corresponding to the regions to be deleted with CRISPR/Cas9) and their FASTA sequences retrieved. A one-exon transcript was generated that spans all the different transcripts modelled by Cufflinks for each of the conditions. Next, we performed pairwise alignments among them, using either the Emboss Emboss Water tool or the Emboss Needle tool^32^, to calculate the local or global sequence identity, respectively. Local comparison was performed for the modelled- transcripts and global comparison for the one-exon transcripts.

### Off-target mutation analysis

DNA regions bearing predicted off-target mutations were amplified by PCR (see primers in Supplementary Table 15) and TIDE analysis^32^. DNA from cells that were not transfected with CRISPR-Cas9 was used as reference. T7-endonuclease assay was performed as follows: a DNA heteroduplex formation was perfomed in a 20 μl reaction containing 300 ng of DNA and 2 μl of buffer 2 (NEB). The conditions for the reaction of the DNA heteroduplex formation are indicated in Ran *et al*.^32^. On DNA heteroduplex formation, digestion was performed with 2 μl of endonuclease T7 (NEB) for 30 min at 37 °C. The digestion was stopped with EDTA and the DNA products visualized in 2% agarose (Nusieve) gels.

### Raw gels and blots

Full gels and blots from the main figures can be found in Supplementary Figure 9.

### Data availability

Raw data from RNA-seq samples was downloaded from GEO (GSE56727). All relevant data are available from the authors.

## Additional information

**How to cite this article:** Montero, J.J. *et al*. Telomeric RNAs are essential to maintain telomeres. *Nat. Commun.* 7:12534 doi: 10.1038/ncomms12534 (2016).

## Supplementary Material

Supplementary Information Supplementary Figures 1-9, Supplementary Tables 1-15 and Supplementary Reference

## Figures and Tables

**Table 1:** Number and percentage of clones obtained for each genotype on deletion of the 20q and Xp loci with the CRISPR-Cas9 system.

	**No. clones**	**No. +/+ (%)**	**No. −/+ (%)**	**No. −/− (%)**
*20q*				
U2OS	55	7 (12.7)	11 (20)	37 (67.3)
HeLa	63	25 (39.8)	34 (53.9)	4 (6.3)
HCT116	47	37 (78)	3 (6)	7 (16)
				
*XpYp*				
U2OS	70	50 (71.4)	16 (22.9)	4 (5.70)
HeLa	49	42 (86.1)	5 (9.8)	2 (4.10)
HCT116	32	22 (68.7)	0 (0)	10 (31.3)
