# Supplementary material for: Telomeric RNAs are essential to maintain telomeres
Source: Nat Commun. 2016 Aug 17;7:12534. doi: 10.1038/ncomms12534 (PMC4992061; doi:10.1038/ncomms12534)
Supplement: Supplementary Information — Supplementary Figures 1-9, Supplementary Tables 1-15 and Supplementary Reference [file ncomms12534-s1.pdf]

Supplementary Figures

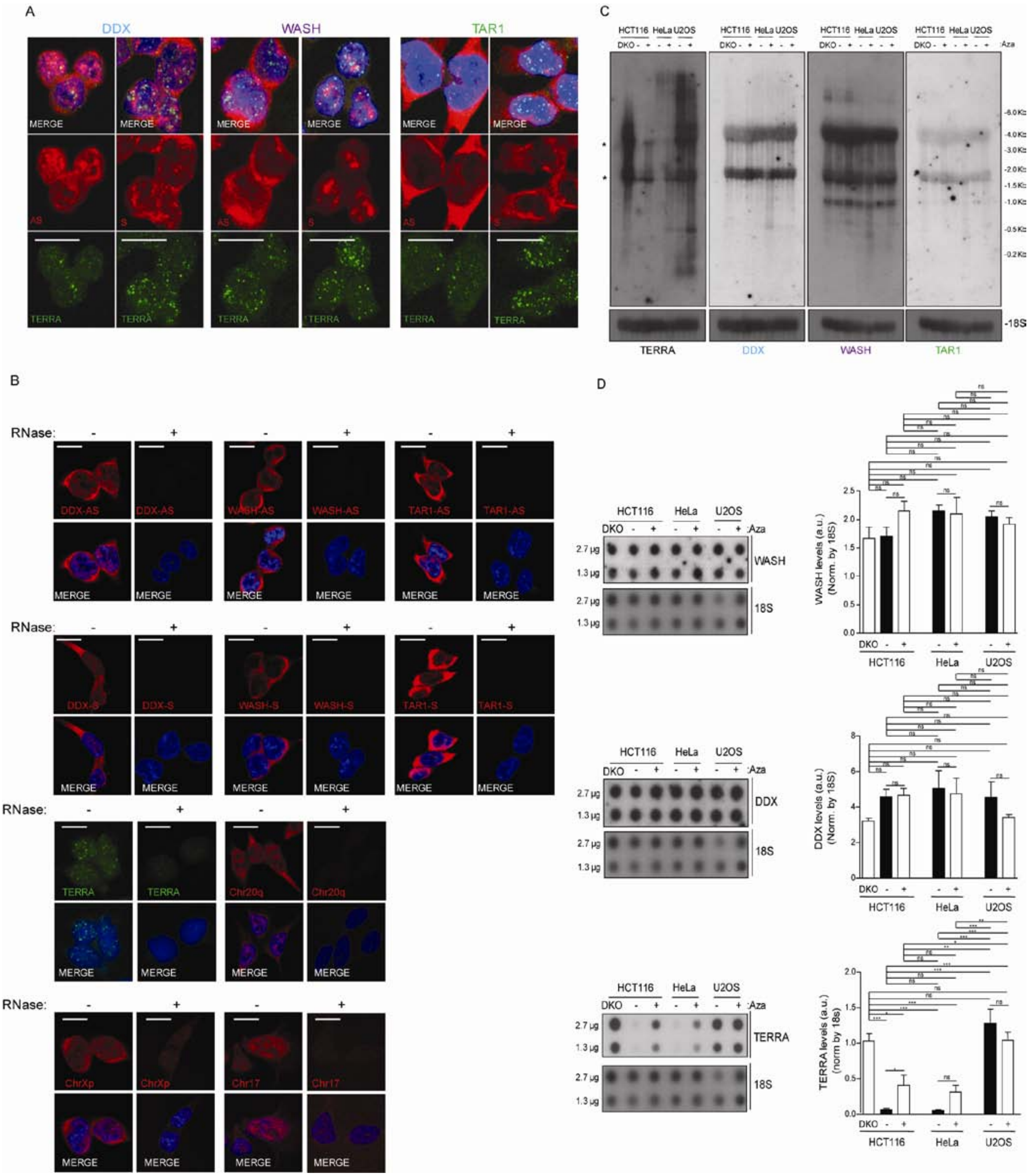

Montero et al._Supplementary Figure 1

Supplementary Figure 1. Transcripts arising from the structurally conserved subtelomeres do not show TERRA features. (A) Confocal microscopy images of double RNA-FISH using probes targeting either TAR1, DDX11L or WASH transcripts (red) and TERRA-UUAGGG track (green). At least 50 nuclei were analyzed in all the cases. AS: probe complementary to the transcript arising from the positive DNA strand; S: probe complementary to the transcript from the negative DNA strand. Scale bar: 10 µm. **(B)** Confocal microscopy images from RNA-FISH with the different RNA-FISH probes in the absence or presence of RNase. Scale bar: 10 µm. **(C)** Northern blotting using ^{32}P-dCTP-labelled probes on total RNA from HCT116 cells DKO (for the DNMT1 and 3b genes) or HCT116 wild-type, HeLa and U2OS treated and untreated with 5′Azacytidin (Aza) for 72 hours. The RNA was used to prepare two identical membranes, in one of them the TERRA probe was hybridized first and, upon stripping, the WASH probe. In the other membrane, the DDX probe was hybridized first and then the TAR1 probe. 18S was included as a loading control. The expected size for DDX transcripts is 1.4-1.6kb and for WASH 1.3-1.8Kb. For TAR1 region, no annotated transcripts have been described so far. *Unspecific band due to cross-hybridization with rRNA 18S and 28S. **(D)** The RNA extracted in (B) was also used for RNA dot-blot to detect either TERRA-UUAGGG track, DDX11L or WASH transcripts; 18S serves as loading control. All the hybridizations were performed on the same membrane one after the other upon stripping. (Graphs) Dot-blot quantification normalized by 18S (mean values±s.e.m., n=3 biological replicates). The Student´s t-test was used for the statistical analysis of the comparison between untreated and Aza-treated cells. One-way Anova with Tukey post test was used for the statistical analysis of the comparison of one cell line with the others (*$p < 0.05$, **$p < 0.01$ and ***$p < 0.001$).

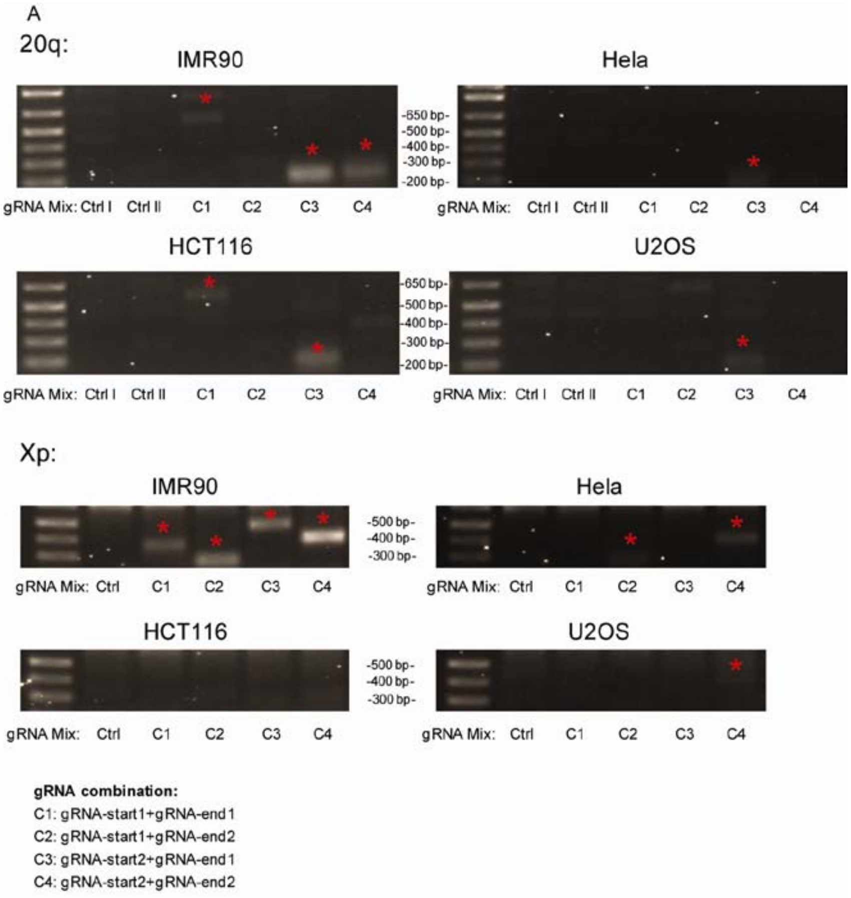

Supplementary Figure 2. Different combinations of gRNAs are able to delete the 20q and Xp loci in different cell lines. IMR90 non-transformed human fibroblasts, HeLa, HCT166 and U2OS cell lines were transfected with different combinations of Cas9-GFP-plasmids containing two different gRNAs to delete 10Kb of the 20q and XpYp region enriched in TERRA transcripts. Two-days upon transfection total DNA was isolated from the different cells lines and PCR using specifics primers to detect the deletion was performed and visualized in ethidium bromide gels. The individual gRNAs used in each combination (C1, C2, C3 and C4) (gRNA mix) are shown below. The primers for the gRNAs synthesis can be found in the **Supplementary Table 5**. In both cases control PCRs (Ctrl I and Ctrl II) using DNA for non treated cells are shown. * DNA amplification found in the treated cells PCR but not in the control PCR.

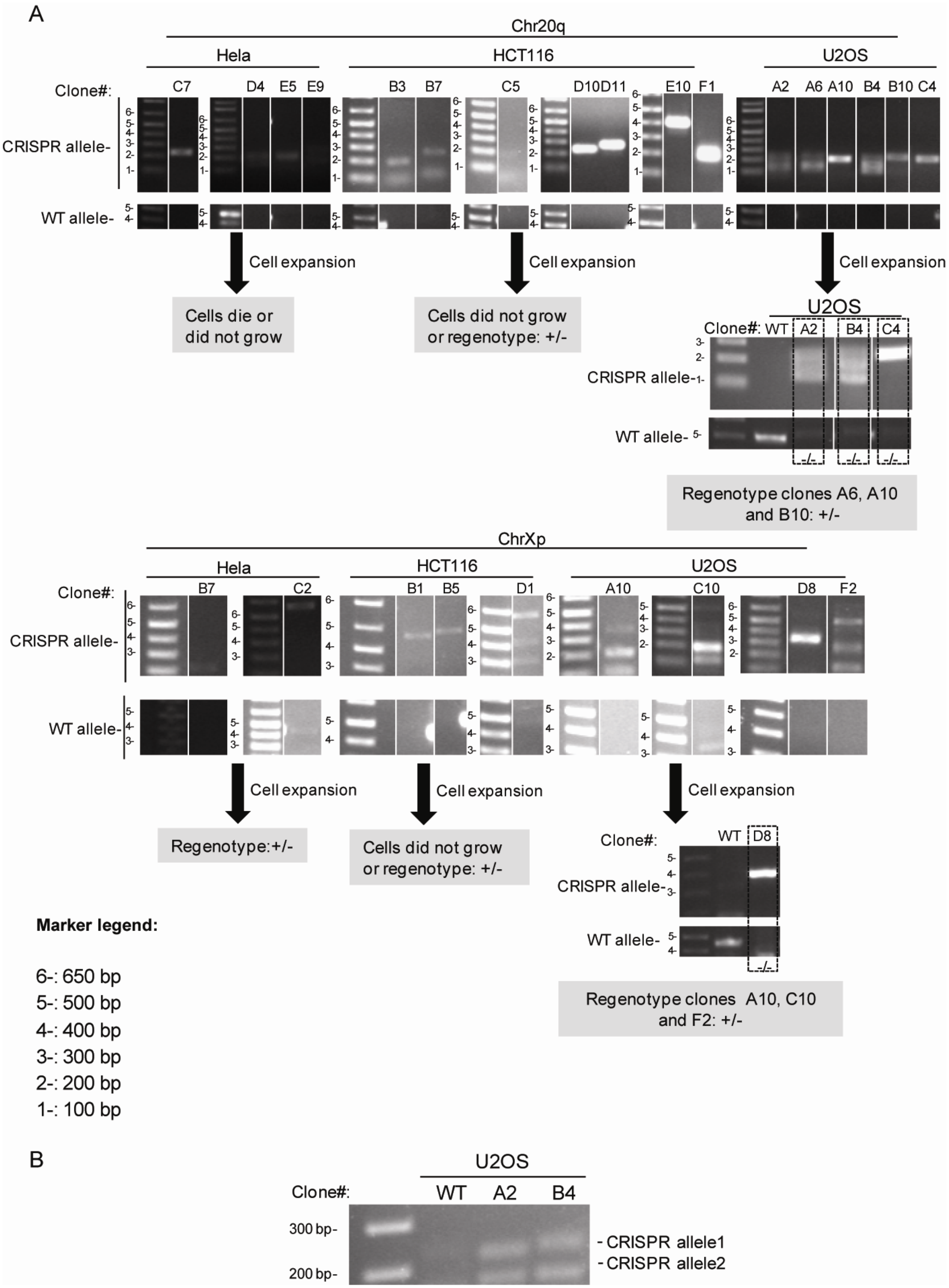

Montero et al._Supplementary Figure 3

Supplementary Figure 3. Regenotyping of false homozygous clones upon expansion. **(A)** Ethidium bromide gels showing the genotype for the CRISPR and wild-type allele in the different clones of the Hela, HCT116 and U2OS cells obtained upon the deletion of the 20q and Xp loci before and after expansion. The primers to detect the deletion were designed to obtain a PCR product of 219 bp for the 20q deletion and of 407bp for the Xp deletion but other sizes can be expected depending on how the CRISPR cut&repair was performed by the cell. The DNA marker legend is shown at the left bottom. Ethidium bromide gels with different expositions are due to the run of the different samples on different days. The white strips in the gels indicate the removal of irrelevant samples from that gel **(B)** Ethidium bromide gels showing the two different CRISPR alleles in the 20q-KO clones A2 and B4

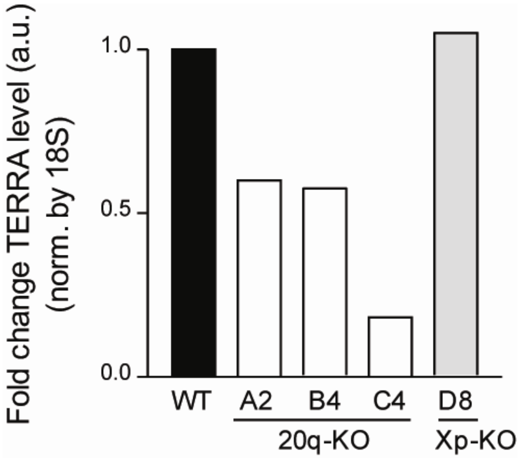

Supplementary Figure 4. Total TERRA levels upon deletion of the 20q locus. Quantification of TERRA signal obtained in the Northern blot from Fig. 3B normalized by 18S. Fold change is shown

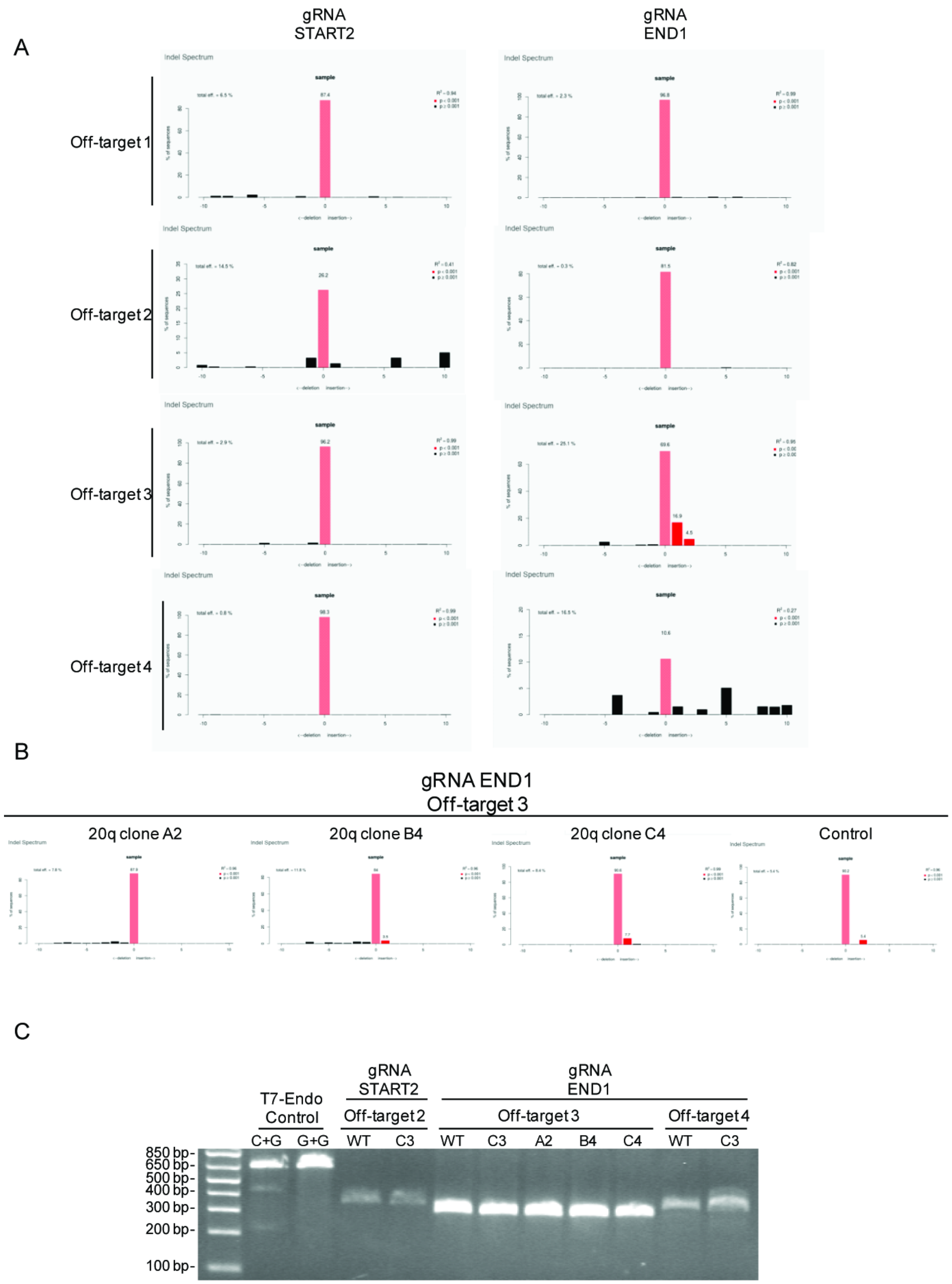

Montero et al._Supplementary Figure 5

Supplementary Figure 5. Off-target mutation analysis in the 20q-TERRA KO cells. (A) TIDE (Tracking of Indels by DEcomposition) analysis to study the four top of targets for the gRNAs (START2 and END1) used to generate the 20q locus deletion. The analysis was performed with DNA pools from either wild type cells or cells bearing the 20q deletion, named C3 (from which the clones A2, B4 and C4 were obtained from). A DNA that did not undergo CRISPR treatment was used as reference. **(B)** TIDE analysis for the off-targets 3, gRNA END1 using DNAs from the 20q-KO clones A2, B4 and C4 and from cells that did not undergo CRSIPR treatment. A DNA from other set of cells that did not undergo CRISPR treatment was used as reference **(C)** T7 endonuclease assay was performed on DNAs from the regions of interest (off-target2/START2, off-target 3/END1 and off-target 4/END1) obtained from pool of cells bearing the 20q deletion (C3) or from the 20q-KO clones. Two identical DNAs except for a C to G mutation in one of them (named C and G) were used as positive control. The ethidium bromide gels shows the different DNAs upon T7 endonuclease digestion.

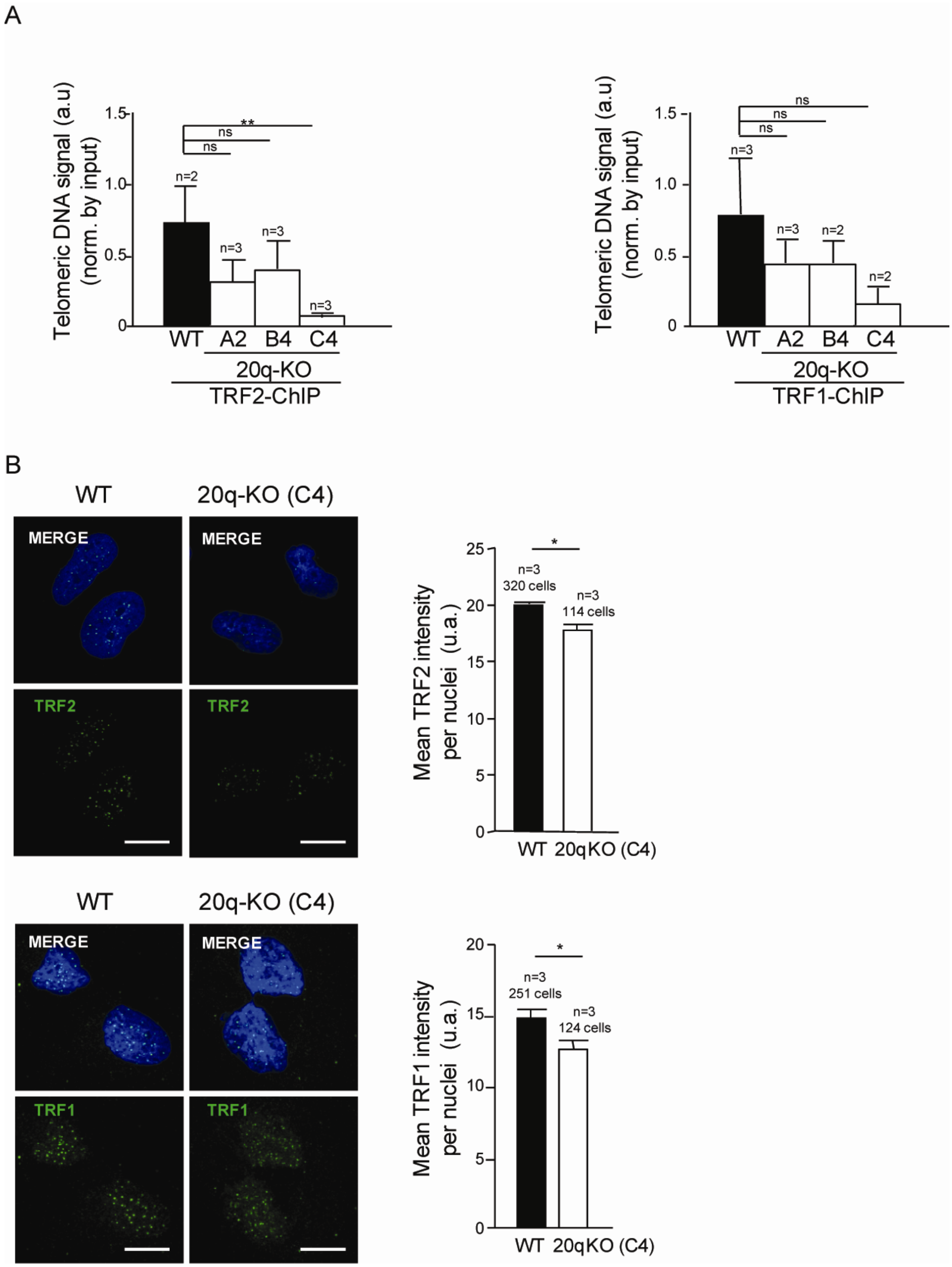

Montero et al._Supplementary Figure 6

Supplementary Figure 6 Detection of shelterins in 20q-TERRA KO cells. (A) Quantification of immunoprecipitated telomeric repeats upon TRF2- or TRF1-ChIP. Values were obtained after normalization to telomeric DNA input and corrected by the differences in the telomere length between the clones and wild-type cells (see Figure 4). Error bars correspond to the 2-3 independent experiments (mean values± s.e.m., n=2-3 independent experiments). One-way Anova with Tukey post test was used for statistical analysis (**$p < 0.01$). **(B)** Representative images of TRF2 and TRF1 immunoflorescence (green) in wild-type cells and 20q-KO TERRA cells (clone C4). Merge images with DAPI are also shown (Graphs). Quantification of the mean fluorescence intensity of TRF1 and TRF2 is represented (mean values± s.e.m., n=3 independent experiments). The Student´s t-test was used for statistical analysis (*$p < 0.05$). Scale bar: 10 µm.

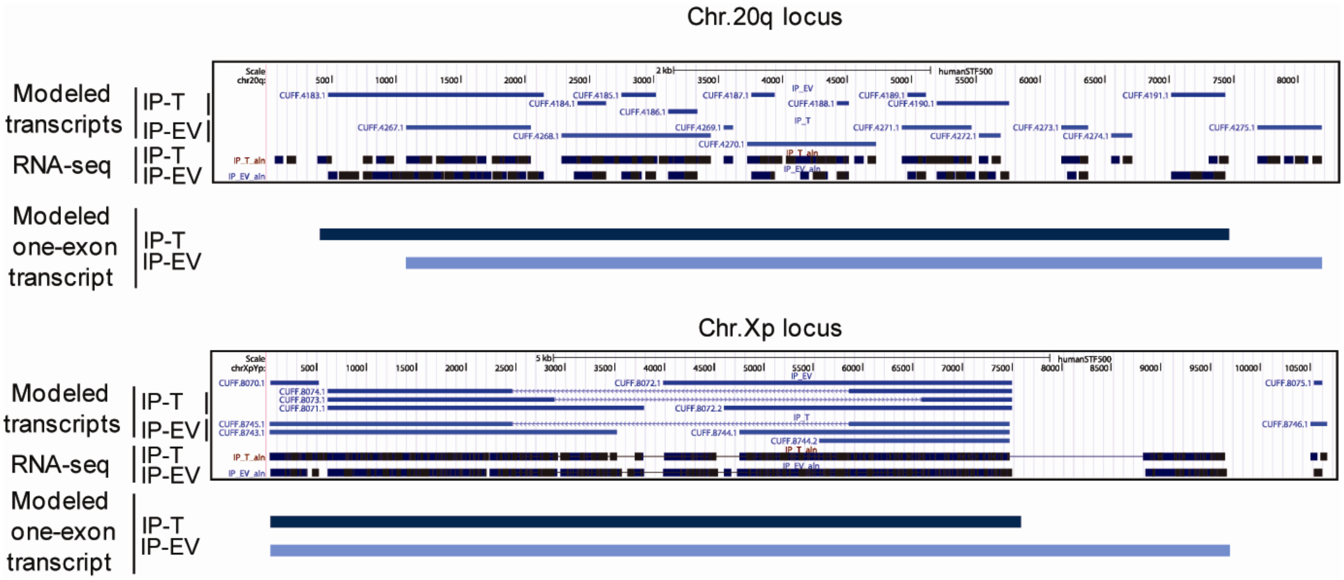

Supplementary Figure 7. Comparison of 20q-TERRA transcripts with Xp-RNAs. Raw data from RNA-seq samples (IP-EV and IP-T)[1] was downloaded and aligned to the human subtelomeric reference genome. Next, transcripts were modeled by Cufflinks in the chr.20q and chr.Xp subtelomeric regions of interest (corresponding to the regions to be deleted with CRISPR/Cas9) and pairwise alignments performed (see Materials and Methods). The snapshot shows (from top to bottom) modeled transcripts in the different IPs, RNA-seq reads (dense format) and the modeled one-exon transcript for each condition

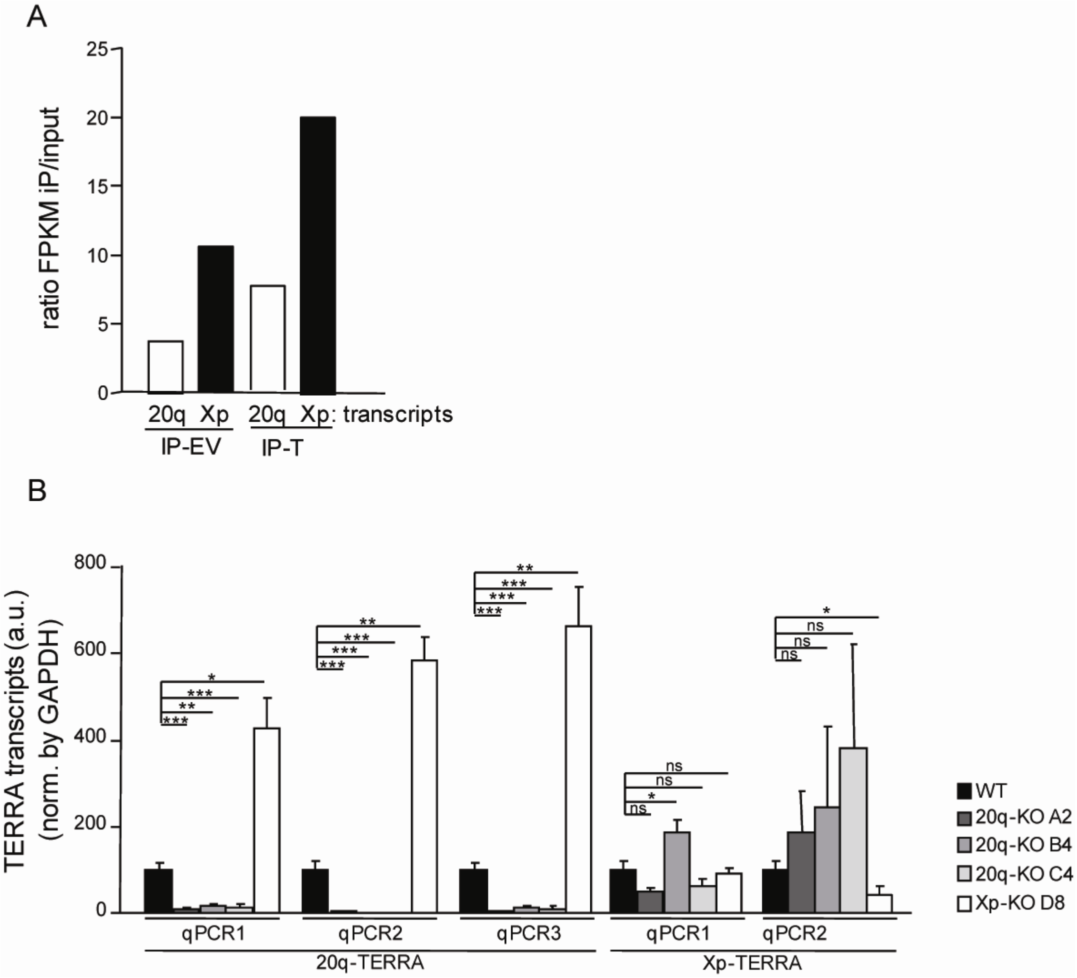

Supplementary Figure 8. Expression analysis of the 20q-TERRA transcripts with respect the Xp-RNAs. (A) Raw data from RNA-seq samples (IP-EV and IP-T) (Porro et al., 2014) was downloaded and aligned to the human subtelomeric reference genome. FPKM (fragments per kilobase of transcript per million fragments mapped) in the chr.20q and chr.Xp subtelomeric regions of interest (corresponding to the regions to be deleted with CRISPR/Cas9) were calculated for each of the IPs and input. (Graph). Quantification of the FPKM in each IP normalized by their corresponding input. **(B)** Detection of the 20q-TERRA or Xp transcripts by PCR using different pair of primers in wild-type cells, 20q-KO clones (A2, B4, C4) and Xp-KO clone (D8). The fold RNA enrichment normalized by GAPDH is shown. Primers 20q/qPCR1-3 are designed within the 20q TERRA locus. Primer Xp/qPCR 1 and 2 are designed within the Xp locus but only the Xp/qPCR2 is designed within the Xp deletion performed in clone D8.

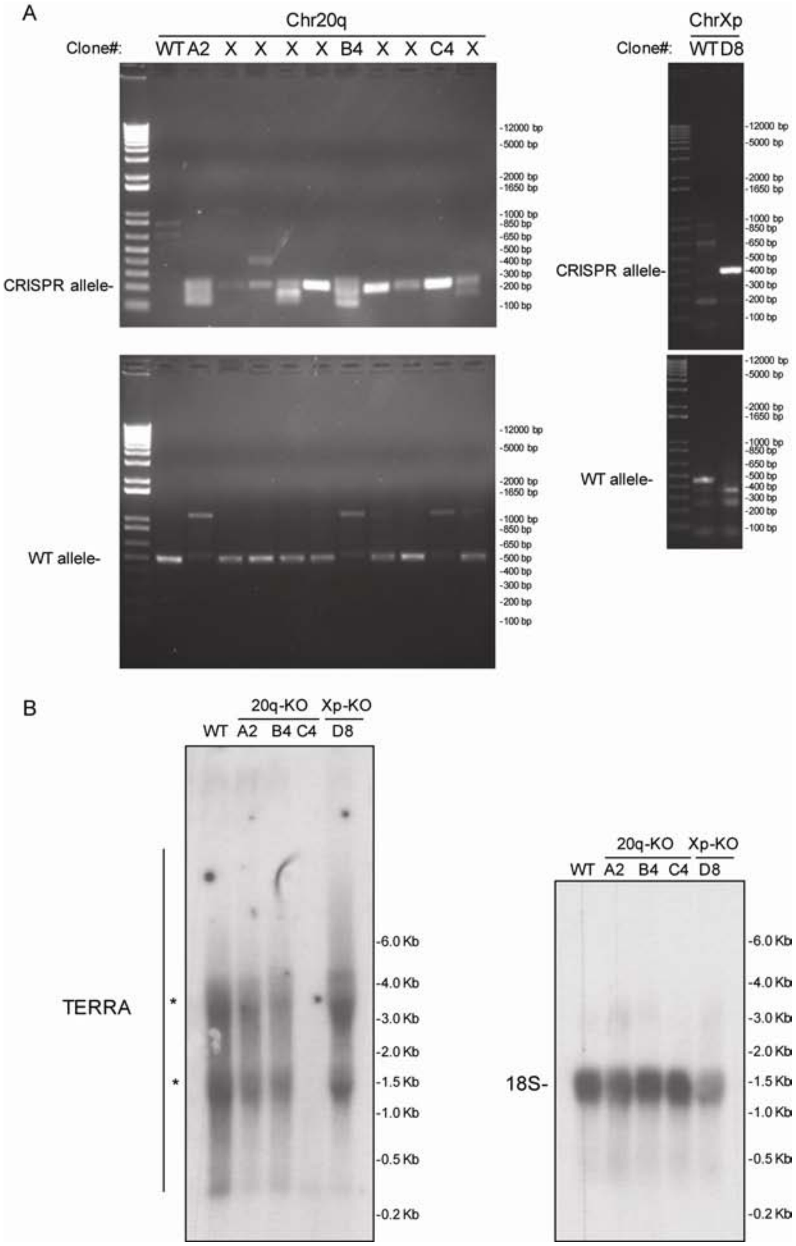

Supplementary Figure 9. Raw gels and blots from Figure 2E and 3B. (A) Ethidium bromide gels showing the CRISPR allele for the deletion of the 20q and Xp loci detected by PCR in different clones of the U2OS cells. The white strips in the gels indicate the removal of irrelevant samples from that gel. ‘X’ are irrelevant samples. **(B)** Northern blotting using 32P-dCTP-labelled probe against TERRA-UUAGGG tract in the U2OS cells WT or KO for the 20q or Xp loci. 18S was included as a loading control. *Unspecific band due to cross-hybridization with rRNA 18S and 28S.

Supplementary Tables

Supplementary Table 1. Chromosome ends amplified by probes against TAR1, DDX and WASH

Probe	Amplified region
TAR1	1q,2q,4q,10q,21q,22q
DDX	1p,2,9p,12p,15q,16p,Xq,Yq
WASH	1p,9p,12p,Xq,Yq

Supplementary Table 2. TAR1 probe specificity recognizing different chromosome ends

	Max score	Total score	Query cove	E value	Ident	Probe origin
Chr1q	697	697	1%	0	100%	1q
Chr2q	697	697	1%	0	100%	2q,4q
Chr5q	675	675	1%	0	99%	2q,4q
Chr6q	675	675	1%	0	99%	10q
Chr9p	481	481	1%	1.00E-138	90%	10q
Chr10q	697	697	1%	0	100%	10q
Chr13q	664	664	1%	0	98%	10q
Chr15q	464	464	1%	1.00E-133	89%	10q
Chr16p	523	523	1%	2.00E-151	92%	10q
Chr22q	697	697	1%	0	100%	22q
ChrXq	483	483	1%	4.00E-139	90%	10q

Supplementary Table 3. DDX probe specificity recognizing different chromosome ends

	Max score	Total score	Query cove	E value	Ident	Probe origin
Chr1p	710	710	1%	0	100%	1p
Chr9p	710	710	1%	0	100%	9p,Xq,Yq
Chr12p	710	710	1%	0	100%	12p
Chr15q	710	710	1%	0	100%	15q
Chr16p	710	710	1%	0	100%	16p
Chr19p	706	706	1%	0	100%	1p
ChrXq	710	710	1%	0	100%	9p,Xq,Yq

Supplementary Table 4. WASH probe specificity recognizing different chromosome ends

	Max score	Total score	Query cove	E value	Ident	Probe origin
Chr1p	701	701	1%	0	100%	1p
Chr9p	697	697	1%	0	100%	9p

Chr12p	701	701	1%	0	100%	12p
Chr15q	682	682	1%	0	99%	1p
Chr16p	645	645	1%	0	98%	1p
Chr19p	676	676	1%	0	99%	1p
ChrXq	701	701	1%	0	100%	Xq,Yq

Supplementary Table 5. Primers for gRNA cloning

Region	Primer	Sequence (5´-3´)
Chr20q	gRNA20q-start1	CACCgTATAGCGGCGGCACGCCGCC
	R-gRNA20q-start1	AAACGGCGGCGTGCCGCCGCTATAc
	gRNA20q-start2	CACCgCAGGCTGGCGCGACGTGCGG
	R-gRNA20q-start2	AAACCCGCACGTCGCGCCAGCCTGc
	gRNA20q-end1	CACCgCCCACCACCTTAGCGGATCA
	R-gRNA20q-end1	AAACTGATCCGCTAAGGTGGTGGGc
	gRNA20q-end2	CACCgAAATTTTACTTCATCGGAGG
	R-gRNA20q-end2	AAACCCTCCGATGAAGTAAAATTTc
ChrXpYp	gRNAXpYp-start1	CACCgCATGGTGGGGACCCGATGCT
	R-gRNAXpYp-start1	AAACAGCATCGGGTCCCCACCATGc
	gRNAXpYp-start2	CACCGCCACCATATCATACTCGGA
	R-gRNAXpYp-start2	AAACTCCGAGTATGATATGGTGGC
	gRNAXpYp-end1	CACCgAACCGGGTGGGTACGTCAAC
	R-gRNAXpYp-end1	AAACGTTGACGTACCCACCCGGTTc
	gRNAXpYp-end2	CACCGGTCTACACCGGCTTCATCC
	R-gRNAXpYp-end2	AAACGGATGAAGCCGGTGTAGACC

Supplementary Table 6. gRNA combinations

Combination#1: gRNA-start1+gRNA-end1
Combination #2: gRNA-start1+gRNA-end2
Combination #3: gRNA-start2+gRNA-end1
Combination #4: gRNA-start2+gRNA-end2

Supplementary Table 7. Primers used to amplify the KO alelle

gRNA combination	Primer	Sequence (5´-3´)
#1,#2,#3,#4	F-20qKOI	CCCGGGTCTGTGTTAAGC
#1,#3	R-20qKOI	GATCAGGGCTGTCTTAATGC
#2,#4	R-0qKOII	TCATCACACCTGCATATACTGT
#1,#2,#3,#4	F-pYpKO	GGCAAAGGGAGCAGTCAT
#1,#2,#3,#4	R-pYpKO	GACGCAACTTCACAGTTACA

Supplementary Table 8. Primers to detect transcripts from the 20q and Xp loci

Region	Primer	Sequence (5´-3´)
20q	F120q-qpcr	CTGGTGCCAGAGTGGATT
	R120q-qpcr	CACCTGTTCTCTTTGTCTGG
	F220q-qpcr	ACATGGGCGATACTCAGG
	R220q-qpcr	CCCACTACTGTGCCTCAA
	F320q-qpcr	GAAGTTGCTGGGTTCTATGG
	R320q-qpcr	ATGGTGCAGACACTGTGG
Xp	F1xp-qpcr	GCAAAGAGTGAAAGAACGAAGCTT
	R1xp-qpcr	CCCTCTGAAAGTGGACCTATCA
	F2xp-qpcr	CTTGAGCTCTCACCACTCAC
	R2xp-qpcr	GCCACGATAGCTTCTTCC

Supplementary Table 9. Chromosomal rearrangements found by CGH array

Chr	Cytoband	Start	Stop	Gain	Loss	Deletion	pval
chr3	p26.3 - p26.1	212.711	6.063.107	0,000000	-0,495877	0,000000	6.11E-97
chr4	q24 - q28.1	102.668.388	126.615.872	0,000000	-0,273520	0,000000	7.73E-09
chr6	p25.3 - p12.3	407.231	51.356.459	0,000000	-0,540169	0,000000	4,900e-324
chr6	p25.3 - p22.2	439.115	26.104.535	0,000000	-0,775369	0,000000	1.51E-80
chr6	p25.3 - p24.3	685.345	9.826.230	0,000000	-0,907387	0,000000	5.65E-07
chr6	p22.1 - p12.3	27.049.061	49.895.377	0,000000	-0,417303	0,000000	6.61E-42
chr6	q27	166.907.999	170.890.108	0,365987	0,000000	0,000000	1.07E-43
chr7	p22.3 - p21.3	707.018	12.258.124	0,313862	0,000000	0,000000	3.10E-92
chr7	p21.3	8.153.319	12.193.158	0,506460	0,000000	0,000000	5.47E-08
chr9	p24.3	329.684	1.411.809	0,000000	-0,866823	0,000000	1.43E-42
chr9	p24.3 - p21.3	1.869.792	23.690.332	0,778509	0,000000	0,000000	4,900e-324
chr9	p24.3 - p24.2	1.869.792	4.357.696	0,517094	0,000000	0,000000	9.67E-11
chr9	p24.2 - p23	4.516.000	11.573.590	1,181,035	0,000000	0,000000	1.82E-62
chr9	p22.1 - p21.3	18.838.925	23.660.030	0,502066	0,000000	0,000000	5.72E-29
chr10	p15.2 - p15.1	3.735.020	4.133.505	0,000000	0,000000	-1,917,017	1.37E-27
chr10	q11.21 - q24.1	44.877.427	97.315.278	0,000000	-0,359404	0,000000	4,900e-324
chr10	q23.1 - q23.33	84.298.588	96.872.423	0,000000	-0,474244	0,000000	2.15E-11
chr12	p11.22 - p11.1	29.329.809	34.345.585	0,000000	-0,401302	0,000000	1.07E-61
chr12	p11.1	33.636.657	34.345.585	0,000000	0,000000	-1,189,862	1.05E-23
chr14	q11.2 - q24.3	20.253.739	78.177.244	0,308504	0,000000	0,000000	4,900e-324
chr14	q11.2 - q21.2	21.697.792	45.083.729	0,411750	0,000000	0,000000	1.20E-17
chr15	q12 - q13.1	27.571.940	28.525.460	0,000000	-0,334935	0,000000	1.24E-09
chr15	q14 - q26.3	38.466.370	98.966.441	0,000000	-0,461232	0,000000	4,900e-324
chr15	q14 - q22.2	39.979.988	60.823.012	0,000000	-0,284430	0,000000	4.02E-65
chr15	q23 - q26.2	71.272.476	96.982.260	0,000000	-0,642954	0,000000	7.53E-74
chr15	q23 - q24.2	72.670.081	75.224.254	0,000000	-0,877269	0,000000	3.38E-12
chr18	p11.32 -p11.21	142.096	13.971.521	0,000000	-0,571498	0,000000	4,900e-324

chr18	p11.32	142.096	293.595	0,000000	0,000000	-1,636,014	1.97E-13
chr18	p11.31 - 11.23	3.954.575	8.055.875	0,000000	-0,786816	0,000000	1.40E-11
chr18	p11.21	11.286.577	13.885.315	0,000000	-0,362484	0,000000	9.21E-10
chr19	p13.3	5.843.077	6.851.877	0,412088	0,000000	0,000000	5.38E-32
chr19	q11 - q13.11	28.272.497	33.976.688	0,000000	-0,490308	0,000000	1.60E-109
chr21	p11.2	9.832.936	9.834.682	0,000000	-0,721174	0,000000	1.30E-36
chr21	q11.2 - q22.13	14.640.392	38.608.514	0,000000	-0,533194	0,000000	4,900e-324
chr21	q22.11 - 22.13	35.210.724	38.608.514	0,000000	-0,811744	0,000000	1.04E-19

Supplementary Table 10. Percentage of identity between the different modeled transcripts in the 20q and Xp loci. IP-EV: IP-sh scramble; IP-T: IP-shTRF2[1]

		Chr.20q transcripts								
	IP-T	**4267.1**	**4268.1**	**4269.1**	**4270.1**	**4271.1**	**4272.1**	**4273.1**	**4274.1**	**4275.1**
Chr.Xp transcripts	**8745.1**	39.3	39.1	53.9	46.3	50.9	36.3	45.1	46.9	37.9
	**8743.1**	37.4	41.2	58.6	38.2	36	47.2	44.2	41.2	40.3
	**8744.1**	40.1	36.4	55.9	35.1	30.4	35.5	35.8	42.1	33.7
	**8744.2**	37.4	36.4	55.9	45.2	45.4	35.5	35.8	42.1	39.5
	**8746.1**	41.4	39.8	38.2	42.5	49.8	38.6	42.4	50.3	39.1

		Chr.20q transcripts								
	IP-EV	**4183.1**	**4184.1**	**4185.1**	**4186.1**	**4187.1**	**4188.1**	**4189.1**	**4190.1**	**4191.1**
Chr.Xp transcripts	**8070.1**	40.9	48.9	39	42.2	38.6	41.4	41.8	43.8	40.7
	**8074.1**	36.5	41.4	40.4	37.1	43.2	82	42.3	52.8	39
	**8073.1**	37	38.4	44.5	36.1	42.6	71.6	45.1	34.4	45
	**8071.1**	43.5	38.4	44.5	36.1	42.6	50.8	39.7	43.5	45
	**8072.1**	37	47.8	40	38.2	43.1	71.6	45.1	34.4	38.4
	**8072.2**	38.6	47.8	40	38.2	43.1	71.6	45.1	34.4	38.4
	**8075.1**	32.8	51.1	37.2	48	54.8	42.4	46.6	41.6	38.2

Supplementary Table 11. Percentage of identity, size (bp) and presence of DNA repeats in the 20q-modeled transcripts with more than 50% homology with the Xp-modeled transcripts. IP-EV: IP-sh scramble; IP-T: IP-shTRF2[1]

	**20q RNAs**	**%Homology with Xp**	**Transcript size**	**Repeats**
**IP-T**	**4269.1**	53-55%	72 bp	LTR
	**4271.1**	50%	546 bp	SINE/LINE
	**4274.1**	50%	167 bp	-

**IP-EV**	**4188.1**	82-71%	90 bp	SINE/LINE
	**4187.1**	54%	185 bp	LTR
	**4184.1**	51%	219 bp	LINE/LTR

Supplementary Table 12. Percentage of Identity between the one-exon modeled transcript from the 20q and Xp loci. IP-EV: IP-sh scramble; IP-T: IP-shTRF2[1]

	**20q-RNAs vs Xp-RNAs**
**IP-EV**	37.70%
**IP-T**	39.20%

Supplementary Table 13. Primers for RNA-FISH and Northern blot probes

**Region**	**Primer**	**Sequence (5´-3´)**
TAR1	F-ProbeTAR1	GCAGAGTTCTTCTCAGGTCA
	R-ProbeTAR1	TGTCATGTGTGCATTAGGAA
	F-T7ProbeTAR1	ccaagcttctaatacgactcactatagggagaGCAGAGTTCTTCTCAGGTCA
	R-T7ProbeTAR1	ccaagcttctaatacgactcactatagggagaTGTCATGTGTGCATTAGGAA
DDX11L	F-ProbeDDX11L	TGTGTGGAAGTTCACTCCTG
	R-ProbeDDX11L	CAGGATGGAAGACAGATTGG
	F-T7ProbeDDX11L	ccaagcttctaatacgactcactatagggagaTGTGTGGAAGTTCACTCCTG
	R-T7ProbeDDX11L	ccaagcttctaatacgactcactatagggagaCAGGATGGAAGACAGATTGG
WASH	F-ProbeWASH	GCCTCTTAAGAACACAGTGG
	R-ProbeWASH	ATCTCTGGGAAAGGACCTG
	F-T7ProbeWASH	ccaagcttctaatacgactcactatagggagaGCCTCTTAAGAACACAGTGG
	R-T7ProbeWASH	ccaagcttctaatacgactcactatagggagaATCTCTGGGAAAGGACCTG
Chr20q	F-ProbeChr20q	CATTGCTGGTGGAAGACAG
	R-ProbeChr20q	GGACCCTCTGTAACAAATGAC
	F-T7ProbeChr20q	ccaagcttctaatacgactcactatagggagaCATTGCTGGTGGAAGACAG
	R-T7ProbeChr20q	ccaagcttctaatacgactcactatagggagaGGACCCTCTGTAACAAATGAC
ChrXpYp	F-ProbeChrXpYp	AACCTGCCGTATCTCACC
	R-ProbeChrXpYp	ACACTCCTGTAGTCCCACCT
	F-T7ProbeChrXpYp	ccaagcttctaatacgactcactatagggagaAACCTGCCGTATCTCACC
	R-T7ProbeChrXpYp	ccaagcttctaatacgactcactatagggagaACACTCCTGTAGTCCCACCT

Supplementary Table 14. Primers for promoter region cloning

**Region**	**Primer**	**Sequence (5´-3´)**
20qPr1	F-0qPr1	CGCGGTACCAAGCCTAAGTGTGGCAAGTC
	R-0qPr1	CCCGCTCGAGCAAGGTGTGTGCTGAGAAAG
20qPr2	F-0qPr2	CGCGGTACCGGAATCATGAAGTATGTGACC

	R-0qPr2	CCCGCTCGAGGGCCTAGTATCCGGAATATACA
20qPr3	F-0qPr3	CGCGGTACCTGTGCGTGTTTCTGTGTC
	R-0qPr3	CCCGCTCGAGGTTCAAGGCCCTCCACAG
20qPr4	F-0qPr4	CGCGGTACCGTGGAGTCAGACCACACG
	R-0qPr4	CCCGCTCGAGTACCAGAAGCTGGAGAAAGG

Supplementary Table 15. Primers for the amplification of DNA regions with predicted off-target mutations

gRNA	Primer	Sequence (5´-3´)
gRNA Start2	F-gS2Off1	TACAAGGTCCGGAGAAGC
	R-gS2Off1	GAGCGGAGGAGGGAGGAC
	F-gS2Off2	TGAGTACCAAGGGTCACCTC
	R-gS2Off2	GGACGGCTGGTTCTCAGG
	F-gS2Off3	GGATGATCTGGGAGAAGC
	R-gS2Off3	GGGAATCTGCAGGACAAAC
	F-gS2Off4	GGGCAGGTAAGGACGTTT
	R-gS2Off4	GCTCCTCACTCCCAAATC
gRNA End1	F-gE1Off1	CTCTAGTTCATGCAGGATGC
	R-gE1Off1	GAGTGAGAAGAGAGCCTTGG
	F-gE1Off2	AATCCAGGAGGCAGAGGT
	R-gE1Off2	CAGGTCAGGGAGTCAACAAC
	F-gE1Off3	TAGGTCACAGAGCAGTAGGG
	R-gE1Off3	GTCCCTAAGGCATTTAGCA
	F-gE1Off4	TTTCCCAACTATTTGCTGAT
	R-gE1Off4	GTTCTGCACCTTATAAACCAG

Supplementary References

1. Porro, A. *et al.* Functional characterization of the TERRA transcriptome at damaged telomeres. *Nat Commun* **5**, 5379 (2014).
